# Systematic discovery of circular permutations across the protein universe using CIRPIN

**DOI:** 10.1073/pnas.2605178123

**Published:** 2026-09-04

**Authors:** Aiden R. Kolodziej, S. Mazdak Abulnaga, Sergey Ovchinnikov

**Affiliations:** ^a^ Department of Biology, Massachusetts Institute of Technology, Cambridge, MA 02139; ^b^ Computer Science and Artificial Intelligence Laboratory, Massachusetts Institute of Technology, Cambridge, MA 02139; ^c^ Department of Radiology, Massachusetts General Hospital, Boston, MA 02115; ^d^Harvard Medical School, Boston, MA 02115

**Keywords:** structural bioinformatics, protein evolution, circular permutation, PDZ domain

## Abstract

Protein structure search has been revolutionized by deep learning methods that can rapidly search massive databases. However, current structure search tools often miss proteins related by topological rearrangements, particularly circular permutation, wherein proteins share highly similar structure but differ in the positioning of their termini. We introduce a circular permutation-invariant graph neural network (CIRPIN) that addresses this limitation through a data augmentation strategy using synthetic circular permutations. We demonstrate that CIRPIN learns representations of proteins that are invariant to circular permutation, enabling it to identify structurally similar proteins within the Structural Classification of Proteins and AlphaFold Cluster Representatives databases. Using CIRPIN, we created CIRPIN-DB, a database of 18.3 million protein pairs highly enriched for circular permutation relationships. Our database contains structures from 845 unique topologies in the CATH Protein Structure Classification database representing the largest and most comprehensive resource of proteins related by a circular permutation assembled to date. Notably, among several novel circular permutants, we find that the PDZ domain—the most commonly inserted domain within multidomain proteins—exists in four distinct circularly permuted forms. Our results establish CIRPIN as a powerful tool to investigate the evolutionary mechanisms underlying circularly permuted proteins.

## Significance

Circular permutation—a phenomenon where proteins fold into a similar 3D shape, yet differ in their termini positioning—presents a fascinating evolutionary puzzle. However, identifying such relationships has remained challenging due to search tools that rely on sequential alignment. We develop a circular permutation invariant graph neural network (CIRPIN) to overcome this problem. Our method identifies proteins related by a circular permutation across databases of solved and predicted structures. CIRPIN reveals previously unrecognized topological diversity within PDZ domains and identifies previously uncharacterized circular permutations across diverse protein families. We anticipate CIRPIN and our accompanying database will serve as an invaluable resource for investigating the evolution of proteins related by circular permutation.

Protein structure prediction tools such as AlphaFold (1) have revolutionized biology by predicting structures for over 200 million proteins (2). To uncover novel folds, identify homologs, and investigate the diversity of protein structures, search tools such as Foldseek (3), TM-Vec (4), and others (5) have been developed. However, these tools are limited in their ability to recover proteins related by structural rearrangements, such as proteins related by a circular permutation.

**Fig. 1.**
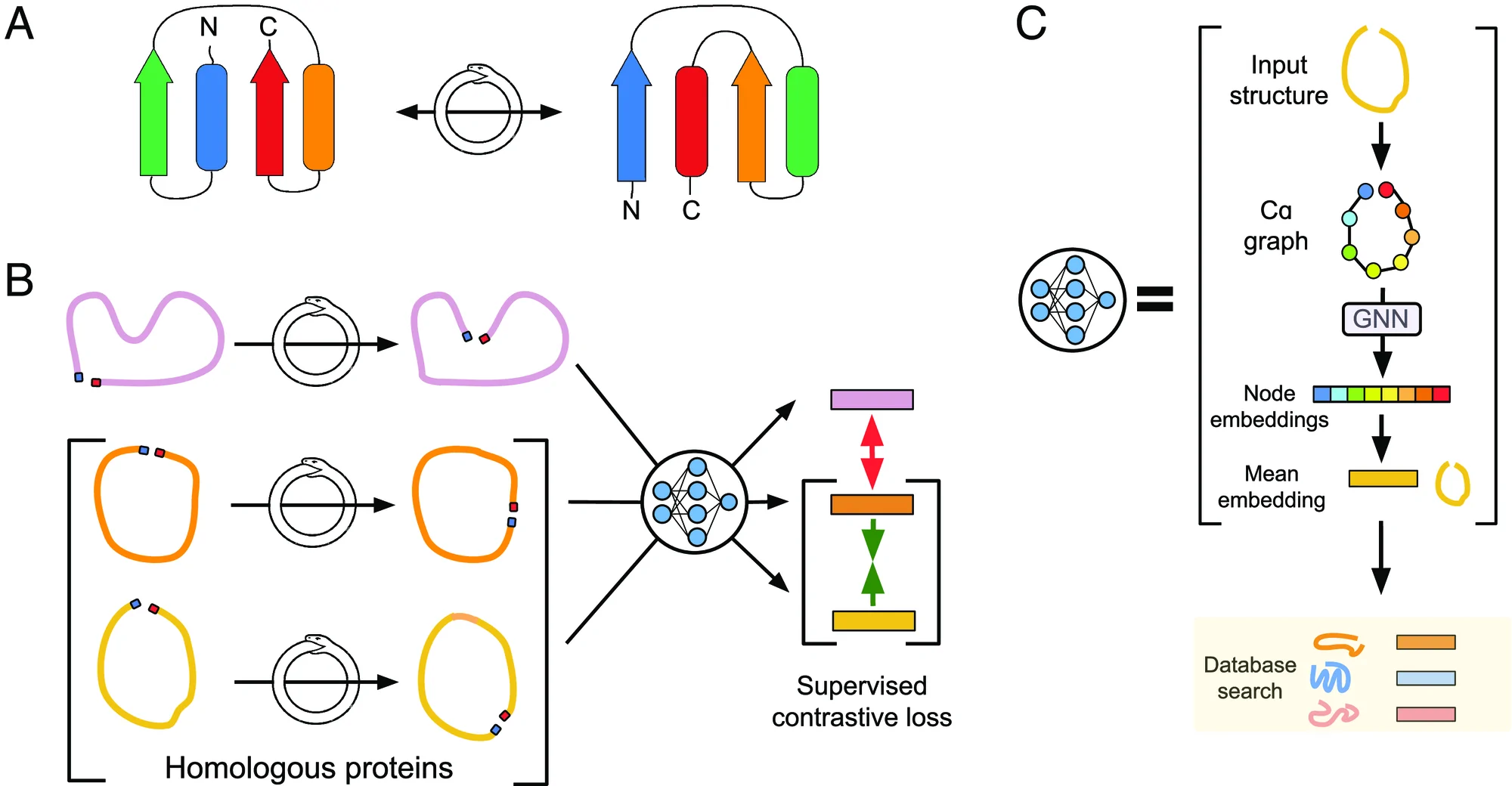
Schematic of CIRPIN model architecture and training. (*A*) Illustrative example showing a circular permutation between two structures. (*B*) CIRPIN training:synCPs of input structures, with N and C termini highlighted in blue and red, respectively, are generated by CIRPIN each iteration. Structures are passed through a graph neural network and a supervised contrastive loss is applied to bring embeddings of proteins in the same SCOPe family (orange and yellow) together while pushing apart proteins from other families (purple). (*C*) Model architecture, adapted from Progres (17). Protein structure is represented as a graph, by extracting C*α* coordinates, which is then passed through an equivariant graph neural network to obtain embeddings. Mean embeddings are then used to search for similar proteins by computing the cosine similarity between the query protein and entries in a precomputed database. Color corresponds to positional information of each node (blue: N terminus, red: C terminus).

Circular permutation (CP), in which the N and C termini are repositioned while preserving the overall protein structure (Fig. 1*A*), has proved useful in a range of engineering and evolutionary contexts. For example, libraries of engineered circular permutants (engCPs) have been used to probe protein folding mechanisms and understand determinants of protein stability (6–8). More recently, CP has been used to design allosteric protein switches (9), protein nanoparticles (10), and protease-sensing Cas9s (11). Consideration of CP can also reveal related proteins that may have otherwise escaped detection through conventional sequence comparison. Notable examples include the evolutionary connection between the bacterial ribosomal protein bL33 and its archaeal/eukaryotic homolog eL42 (12), as well as a CP discovered between two carbohydrate-binding proteins (13). However, these discoveries arose from focused investigations of individual proteins and their known homologs rather than from systematic searches. This highlights the need for sensitive methods to search for circular permutants and investigate their prevalence in nature.

Identifying proteins related by a CP is a challenging task, requiring tools that can both detect a CP and distinguish them from simpler cases of structural or sequence similarity where order has remained largely unchanged. A few methods have been developed for detecting circularly permuted proteins; however, prior work has been limited to explicit alignment via dynamic programming which is prohibitively slow for large-scale comparisons (14, 15). Another method, CE-CP (16), facilitates proper alignment of circularly permuted proteins and is useful for comparing specific cases, but does not provide a metric for distinguishing cases of CP from instances of sequential structural similarity, limiting its use in high-throughput searches.

A key issue with current search tools is their reliance on sequential alignment. Aligning proteins from N to C termini is problematic for comparing proteins with nonsequential relationships, such as circular permutants. One recent structure search method, Progres (Protein Graph Embedding Search) (17), leverages a graph neural network (18) architecture to learn an embedding space for protein structure that can be subsequently used for search. While Progres trains on structure and forgoes explicit alignment, it suffers from the same drawback since it represents protein structure with positional information corresponding to the N and C termini locations (17).

One approach to identifying CPs would be to train a model like Progres on a database of circular permutants; however, as we discover, current databases are small and limited in the diversity of CPs they contain. To address this shortcoming, we devised a synthetic circular permutation operation to generate synthetic circular permutations (synCPs). This operation provides insight into the current limitations of structure search tools and provides a means of imbuing models with invariance to circular permutation. Building on the work of the Progres model (17), we use synCPs to train a circular permutation invariant graph neural network, (CIRPIN) (Fig. 1 *B* and *C*). We then develop a comparative framework, contrasting CIRPIN embeddings to those of a similarly trained, but non-CP aware model (Progres), to identify proteins related by a circular permutation.

Notably, this dual-model approach uncovers multiple PDZ domain topologies related to one another by a CP. Leveraging Progres/CIRPIN’s speed, we perform a search over the AlphaFold Cluster Representatives Database (AFDB-ClustR) (19) and uncover thousands of novel CP relationships.

## Defining Circular Permutation.

Historically, a pair of proteins has been defined as related by a circular permutation if the N-terminal region of one protein has significant sequence similarity to the C-terminus of the other and vice versa (20–22). However, several previously reported cases of proteins related by a circular permutation do not fit this strict sequence-based definition. For example, the enzyme polyhydroxybutyrate depolymerase is reported to be a circular permutation of the canonical *α*/*β* hydrolase fold, despite sequence similarity of only 28% after accounting for the circular permutation (23). In other work, two antitoxin domains lacking sequence similarity were discovered to be related by a circular permutation when comparing the two structures (24). The authors note that these two domains may have evolved independently, converging to a similar structure. In either scenario, the circular permutation refers to a structural relationship, not a sequence-level relationship.

In order to better reflect the usage of “circular permutation,” we propose a definition based on a circular permutation operation (CPO), herein represented by the ouroboros, an ancient symbol of rebirth. Here, a CPO refers to the hypothetical linkage of the N and C termini of a protein with an arbitrary linker, followed by cutting the circularized protein at a location along the chain that introduces new N and C termini positions. If applying this operation to a protein’s structure would result in the same fold (same connectivity of secondary structural elements and arrangement in 3D space) as another protein, these proteins are related by a circular permutation and can be considered circular permutants of one another (Fig. 1*A*). Importantly, defining a circular permutation in this way makes no assumptions about the evolutionary mechanism(s) that give rise to a pair of circular permutants. Hence, our use of the term circular permutation (CP) should not be taken to imply homology. Elucidating the evolutionary history of a pair of circular permutants requires consideration of structure and sequence, as well as explicit tracing along a phylogenetic tree, to disentangle convergent vs. divergent evolution.

## Results

We first assess the ability of Progres (17) and an explicit structural alignment tool, TM-align (25), to find proteins related by a CP. Using our defined synCP, we probe the dependence of these two methods on positional information and find they have a limited ability to detect known CPs. We then compare our model, CIRPIN, against Progres on retrieving known example CPs from the literature. Subsequently, we use CIRPIN to identify novel CPs in the Structural Classification of Proteins - Extended (SCOPe) and the AFDB-ClustR databases. Finally, we demonstrate the learned embeddings of CIRPIN capture structurally meaningful features and CIRPIN can be additionally used to recover similar proteins obscured by other structural changes, such as secondary structure rewiring and insertions.

### Probing Structural Alignment Methods Using SynCPs.

Given that embeddings across various lengths are averaged in Progres, instead of aligned, we hypothesized Progres embeddings of protein structure might be invariant to sequential perturbation, such as CP, and could be used to identify proteins related by a CP. To investigate this idea, we evaluated whether Progres can detect proteins related by a CP within the SCOPe database (clustered at 40% sequence identity) (26).

**Fig. 2.**
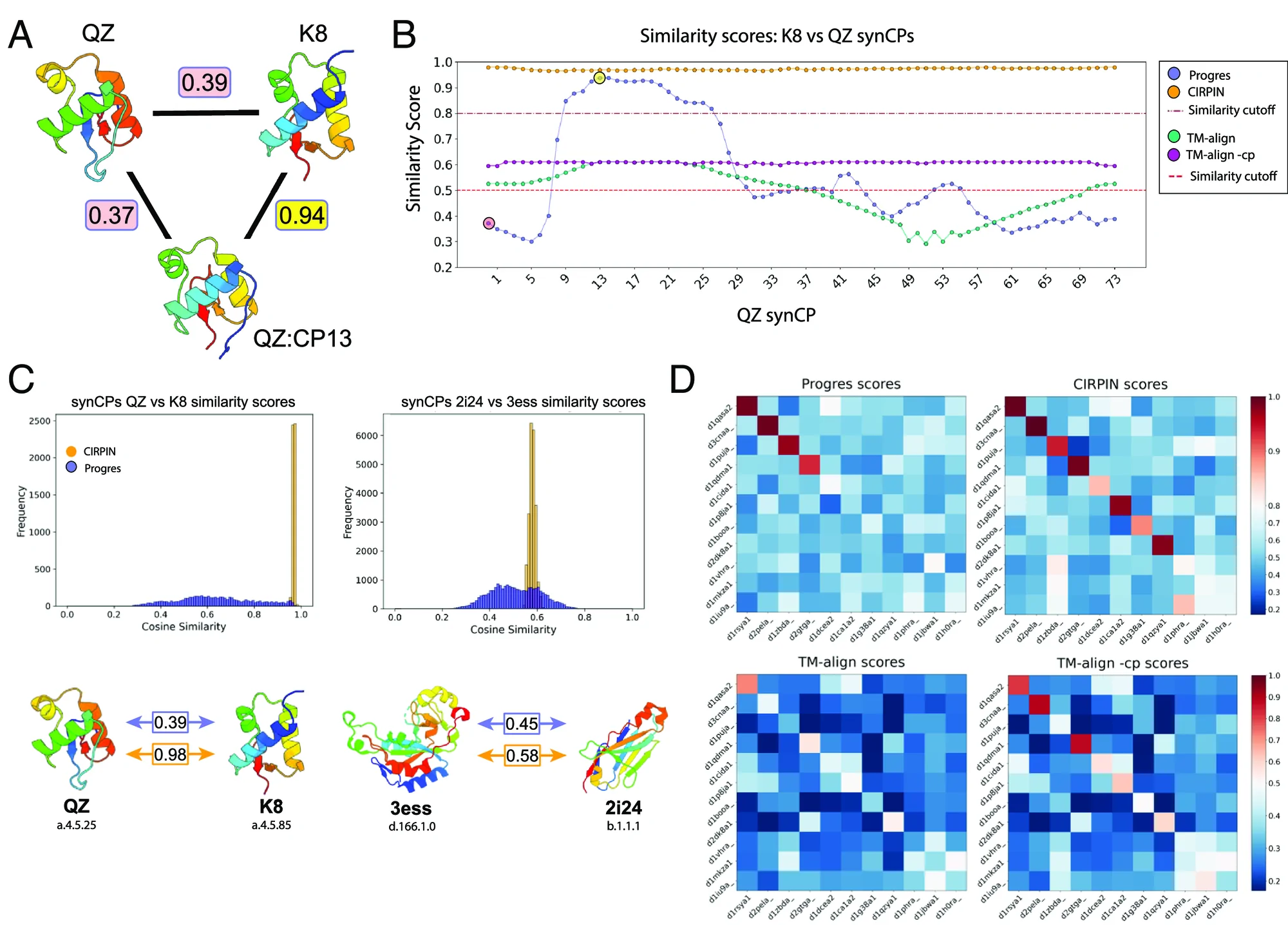
Evaluation of structure search methods on retrieval of proteins related by a circular permutation. (*A*) Scores between QZ, K8, and the 13th synCP of QZ. Progres scores are highlighted in yellow (high) or red (low). Structures are colored from N (blue) to C terminus (red). (*B*) Similarity scores between every synCP of QZ vs. unpermuted K8. Horizontal lines mark the similarity cutoff score for Progres/CIRPIN (0.8) and TM-align (0.5) (*C*) Invariance of CIRPIN to sequence reordering. Plots display scores from an all-vs.-all comparison of query synCPs vs. target synCPs. For a pair related by a CP (QZ and K8), CIRPIN identifies the pair as highly similar. For unrelated pairs, (3ess and 2i24) CIRPIN does not find similarity. In both cases, Progres scores vary widely depending on positional reordering. Scores between unpermuted original structures from the two models shown below. SCOP(e) concise classification string (sccs) shown below structures, indicating *class.fold.superfamily.family*. (*D*) Benchmark set of verified pairs of circular permutations arranged such that scores for pairs of CPs follow the diagonal of the heatmap. Heatmap colors are centered at the similarity cutoffs of 0.5 and 0.8.

As a representative example, we focused our attention on the winged helix–turn–helix (wHTH) fold, a widely conserved DNA-binding domain found in transcription factors across the tree of life (27). Previous structural characterization of a polysaccharide biosynthesis protein from *Clostridium acetobutylicum* revealed the presence of a circularly permuted wHTH (28). These two structures are also present in the SCOPe database as the rpc34 subunit in RNA polymerase III from mouse, d2dk8a1 (hereafter referred to as “K8”), and as the human methionine aminopeptidase d1qzya1 (hereafter referred to as “QZ”). Embedding these two domains using Progres and scoring their similarity via cosine similarity returned a low score of 0.39 (Fig. 2*A*), which following a previously defined similarity threshold of 0.8 (17), indicates no significant structural similarity.

We hypothesized that creating a synCP of a query structure such that the node ordering aligns to the amino acid order of a circularly permuted target protein would result in detection by Progres without any retraining of the model. To test this idea, we generated all possible synCPs for query QZ and scored them against the target K8. The results show a wide range in scores that is solely dependent on amino acid ordering (Fig. 2*B*). As predicted, some synCPs of QZ now score highly against K8 (Fig. 2*B*). The highest scoring structure was QZ permuted at position 13 and aligns well to K8 (Fig. 2*B*).

We also investigated if TM-align could identify similarity between these two proteins. As shown in Fig. 2*B*, although TM-align found similarity between the unpermuted two structures, it struggled to do so for many synCPs (33 out of 73). When an optional -cp alignment parameter was enabled, the resulting scores are unaffected by a CP and are all above the structural similarity cutoff of 0.5 (Fig. 2*B*). However, it is well established that TM-align and related tools are too slow to use on the size of today’s structural databases. For example, it has been estimated that using TM-align to calculate an all-vs.-all comparison of 100 million structures would take approximately 10 millennia on a 1,000 core cluster (3). This issue is further exacerbated by running TM-align with -cp enabled, which is approximately 1.5 times slower than running TM-align.

Next, we generated all synCPs for both the query and target and scored them in an all-vs.-all manner, resulting in 5,920 comparisons for QZ and K8. Plotting the Progres scores of all possible synCP comparisons produced a wide distribution of scores [Fig. 2*C* (blue)], indicating that the score between two proteins can vary greatly, depending solely upon the amino acid order. We found this remained true for two random proteins unrelated by CP and have different SCOPe class designations (Fig. 2*C*). We also investigated whether TM-vec (4) has a similar limitation and found that scores between synCPs can vary considerably (*SI Appendix*, Fig. S1).

Based on these results and motivated by Progres’s speed relative to TM-align, we investigated how Progres could be modified to achieve circular invariant protein representations. To this end, we developed CIRPIN.

### Benchmarking CIRPIN.

We hypothesized that by training the Progres graph neural network (Fig. 1*B*) on synthetic circular permutants (synCPs), the model would learn embeddings such that circular permutants are scored as similar. Furthermore, such a model would become less dependent on positional information and instead learn interacting structural motifs to more accurately embed proteins in a shared latent space. We first considered removing positional information entirely, but as shown previously (17), this drastically reduces the model’s ability to accurately retrieve homologous proteins.

After training CIRPIN, we used our model to embed QZ and K8 and found CIRPIN correctly scored them as highly similar (Fig. 2*C*). We then compared the distribution of CIRPIN and Progres scores (Fig. 2*C*) for all synCPs of QZ and K8. The narrow distribution of CIRPIN scores indicates that circularly permuting the positional information, by generating synCPs, has little effect on the score calculated by CIRPIN, whereas it greatly alters the scores output by Progres. These observations held true when comparing two unrelated proteins (Fig. 2*C*).

### Retrieval of Homologous Proteins.

One concern with training on synCPs is that CIRPIN might overgeneralize and identify similarity in structurally dissimilar proteins. As a sanity check, we evaluated CIRPIN’s ability to recover domains from the same SCOPe fold, superfamily, and family using the test set constructed by Progres. This set comprises 400 randomly selected domains from the Astral 2.08 40% sequence identity dataset, with no training set domains sharing ≥30% sequence identity with these test cases. Table 1 shows that CIRPIN achieves recovery scores comparable to Progres (higher is better). We expect CIRPIN to perform slightly worse than Progres on this task, since CIRPIN is trained, by means of our synCP operation, to learn similarity between proteins that are not already neatly classified into the same family by SCOPe.

**Table 1.** Comparison of ability to retrieve homologous proteins from SCOPe

Model	Fold	Superfamily	Family	SCOPe database run time (all vs. all)
CIRPIN	0.166	0.692	0.865	154 s
Progres	0.177	0.706	0.877	155 s

To evaluate their generalizability beyond SCOPe, we benchmarked Progres and CIRPIN on the Evolutionary Classification of Domains (ECOD) database (29), which provides a robust dataset for homology recovery. We sampled 1,000 ECOD H-level groups where H-level groupings contain proteins with strong support of homology (29). Pairwise all-vs.-all comparisons were performed within each group. CIRPIN recovered homologous pairs missed by Progres in 407/1,000 groups (41%), while Progres outperformed CIRPIN in only 80/1,000 groups (8%) (*SI Appendix*, Fig. S2). Analysis of pairs with the highest CIRPIN/Progres differences revealed pairs related by a CP (*SI Appendix*, Fig. S2), suggesting CIRPIN’s improved recovery of homologous relationships.

### Recovering Circular Permutations in SCOPe.

An ideal measure of CIRPIN’s performance at recovering protein pairs related by a CP would be testing using a benchmark dataset consisting of structurally diverse pairs of circular permutants. Unfortunately, no such set has been established in the field, with researchers relying on only a few well-studied domains, such as the concanavalin A lectin domain (30–32) to test their methods. To address this issue, we performed a thorough literature review of reported circular permutants and constructed a test set of 11 protein pairs which were aligned in PyMOL (33) and verified visually to be related by a CP, with their termini at different locations relative to one another. It is important to note that some circular permutations are more “severe” than others. In severe cases, the termini are located far away from the other protein’s termini locations, in subtler cases of CP the termini locations may have moved relatively little. Thus, this dataset is heterogeneous in regard to the degree of circular permutation.

We scored this database using TM-align (*Methods*): TM-align recovered 3 out of 11 and TM-align -cp recovered 7 out of 11 (Fig. 2*D* and Table 2). For retrieval using Progres or CIRPIN, a cosine similarity above 0.8 corresponds to a successful recovery (17) (Fig. 2*D* and Table 2). Of the 11 test domains, Progres recovered 4 and CIRPIN recovered 9. However, two of these pairs are grouped within the same SCOPe family, and so during training the embeddings of these structures would have been pushed closer to one another. Since we seek to determine how well the two models perform at recovery in instances where SCOPe does not classify circular permutants in the same family, the adjusted recovery rates are 2 out of 9 for Progres and 7 out of 9 for CIRPIN (Fig. 2*D* and Table 2). The two pairs CIRPIN was unable to identify were also missed by TM-align -cp, indicating that other structural changes, in addition to CP, obscure the structural similarity between these proteins. For example, a careful visual comparison of d1iu9a and d1h0ra revealed that a region composed of a long beta sheet in d1h0ra is present as a shorter beta sheet and alpha helix in d1iu9a.

**Table 2.** TM-align and embedding similarity scores for benchmark CP pairs

PDB A	SCOPe A	PDB B	SCOPe B	TM	TM-CP	Progres	CIRPIN
d1rsya1	b.7.1.2	d1qasa2	b.7.1.1	0.722	0.811	0.987	0.991
d2pela_	b.29.1.1	d3cnaa_	b.29.1.1	0.471	0.891	0.989	0.997
d1zbda_	c.37.1.8	d1puja_	c.37.1.8	0.326	0.477	0.973	0.939
d2gtga_	a.64.1.1	d1qdma1	a.64.1.2	0.555	0.875	0.934	0.987
d1dcea2	b.7.4.1	d1cida1	b.1.1.1	0.460	0.567	0.790	0.855
d1ca1a2	b.12.1.3	d1p8ja1	b.18.1.2	0.499	0.622	0.570	0.978
d1g38a1	c.66.1.27	d1booa_	c.66.1.11	0.361	0.503	0.688	0.887
d1qzya1	a.4.5.25	d2dk8a1	a.4.5.85	0.526	0.596	0.389	0.979
d1phra_	c.44.1.1	d1vhra_	c.45.1.1	0.295	0.462	0.377	0.805
d1jbwa1	c.59.1.2	d1mkza1	c.57.1.1	0.368	0.491	0.451	0.772
d1h0ra_	c.23.13.1	d1iu9a_	c.78.2.1	0.374	0.374	0.737	0.755

### Prevalence and Discovery of Circular Permutations in SCOPe.

**Fig. 3.**
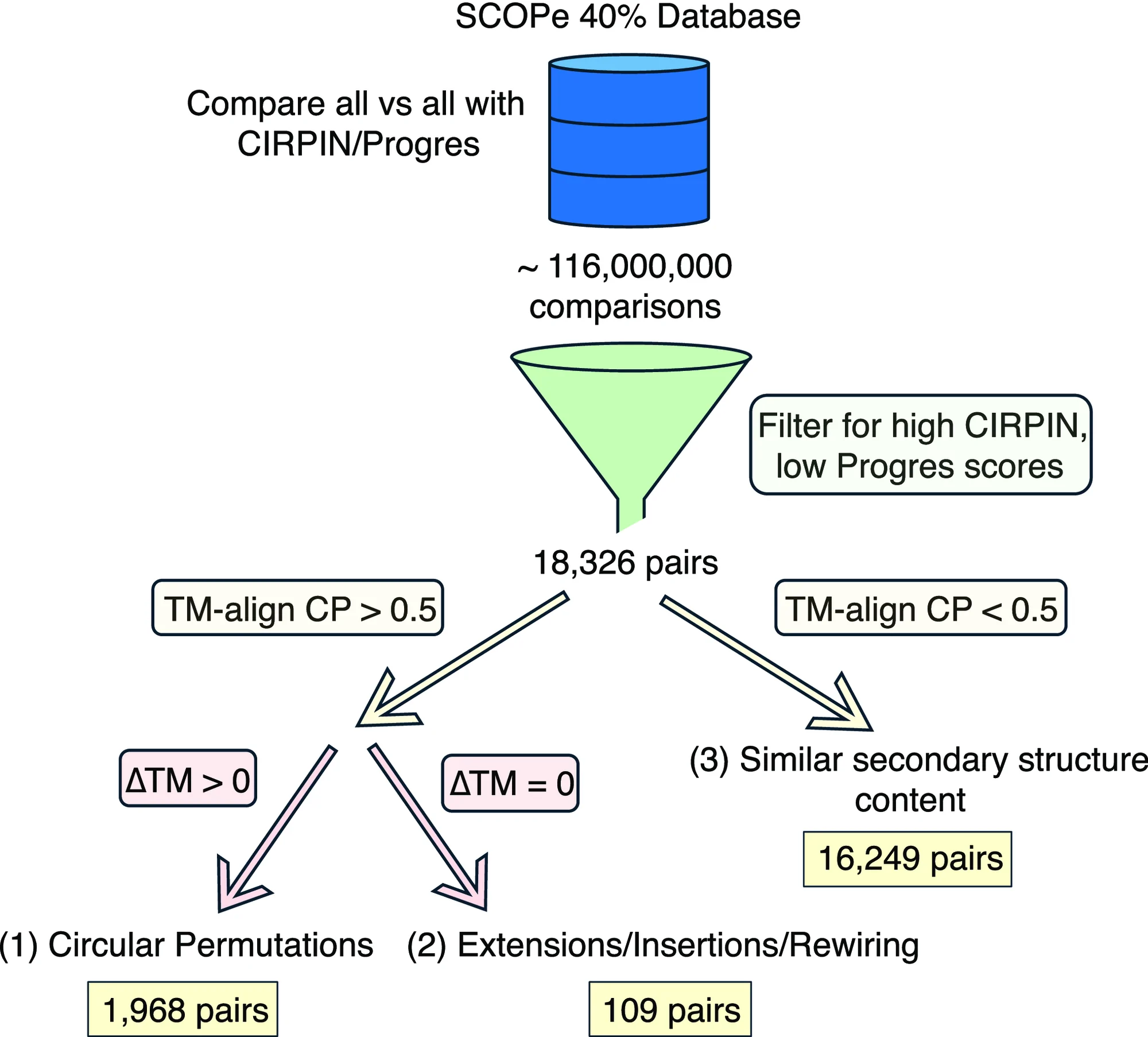
Schematic for identifying proteins in SCOPe related by a circular permutation, rewiring, or extensions/insertions. Cutoffs used for Progres and CIRPIN scores were 0.6 and 0.9, respectively. ΔTM is the difference between TM-align and TM-align -cp scores.

We embedded the entire Structural Classification of Proteins - Extended (26) (SCOPe) database (clustered at 40% sequence identity, ∼15.2 k structures) using both CIRPIN and Progres models, then performed an all-vs.-all comparison. To identify pairs related by a CP with high confidence, we developed the search pipeline outlined in Fig. 3*A*. We use Progres and CIRPIN as a rapid filter for identifying putative CPs and then use TM-align as a sensitive alignment method to further classify these hits.

To set model thresholds, we searched the test set of SCOPe against the entire SCOPe database using Progres and CIRPIN (clustered at 40 % sequence identity) and plotted the top 200 matches according to TM-align. We found that Progres displayed an area of high density below 0.6 that corresponds to TM scores below 0.5 and CIRPIN displayed an area of high density above 0.9 that corresponds to TM scores above 0.5 (*SI Appendix*, Fig. S3).

Because our approach targets cases where CIRPIN and Progres disagree, we sought thresholds where CIRPIN confidently identifies structural similarity and Progres suggests none. We found that 95.0% of pairs with CIRPIN >0.9 had TM-score >0.5, and 70.7% of pairs with Progres <0.6 had TM-score <0.5. We therefore adopted CIRPIN >0.9 and Progres <0.6 as cutoffs for detecting divergent predictions between the two models (*SI Appendix*, Fig. S3).

Following filtering, we obtained 18,326 protein pairs with high Progres/CIRPIN score differences. When aligned using TM-align (25) with consideration of CP, most of these pairs (16,249 or ∼89%) had TM-scores below 0.5, suggesting different folds and likely excluding cases of CP. Further analysis revealed that pairs in this bin tend to have similar secondary structure content. Specifically, pairs in this bin share the same SCOP class ∼80% of the time, a fourfold enrichment over the ∼20% baseline chance of two random proteins sharing the same SCOPe class.

Using the difference between TM-align with and without consideration of CP (*Methods*) we identified 1,973 pairs likely related by a CP. After filtering out domains occurring in multiple pairs, there are 1,204 unique structures. To better account for structural redundancy, we took a similar approach to ref. 34 and clustered these structures using Foldseek (19) which resulted in 193 clusters. Since this result depends on CIRPIN/Progres thresholds, we made modest changes (±0.05) to these cutoffs and recalculated the number of clusters (*SI Appendix*, Fig. S4). This gave a range of 193 to 304 clusters. Clustering the entire SCOPe40 using the same parameters resulted in 4,357 clusters.

Using clusters, which capture structural redundancy in SCOPe 40%, we estimate the rate of circular permutation in SCOPe to be between 4.4% to 7.0%. Importantly, this estimate is substantially lower than previous estimates of CP prevalence, which have ranged broadly from 9 to 36% (14) and were based off a database constructed using CPSARST (14, 22).

### Structural Redundancy and Bias in the Circular Permutation Database (CPDB).

To investigate the discrepancy between our estimate of CP prevalence and previous reports, we examined the Circular Permutation Database (CPDB) (22), consisting of 4,169 pairs of circular permutants reported to be nonredundant.

We identified several factors that contribute to the much higher rate of CP reported by other groups. The first is structural redundancy present in the CPDB: We found that ∼20% of pairs (815/4,169) in the CPDB contain a structure classified in SCOPe as concanavalin-like (*SI Appendix*, Fig. S5). Since concanavalin (PDB: 2CNA), discovered in 1979 (21), is a well-studied example of a circularly permuted fold, many homologous structures have been deposited in the PDB, resulting in overrepresentation of the structure in the CPDB. Second, we noticed pairs with low TM-scores, even after accounting for CP (*SI Appendix*, Fig. S6), suggesting that the manual refinement process used in constructing the CPDB did not eliminate all false positives.

Last, we noticed several pairs with near perfect TM-scores (>0.9) after accounting for CP (*SI Appendix*, Figs. S5 and S7) which were not present in our database. We found that these pairs are not naturally occurring CPs, but are rather a result of including engineered circular permutants present in the PDB. We identified two SCOPe families, comprising ∼5.6% of CPDB pairs, which contain engineered CPs, though additional cases may exist (*SI Appendix*, Fig. S5). Importantly, we noted that engCPs are classified in the same SCOPe family as one another. Since the CIRPIN/Progres loss function optimizes for intrafamily similarity, Progres and CIRPIN scores between engCPs are identical (*SI Appendix*, Fig. S7) and because our method filters for pairs with large CIRPIN/Progres score differences, engCPs are excluded from our database.

After applying the same quality control metrics we used for identifying CPs, the CPDB consists of 1,467 pairs of CPs (*SI Appendix*, Fig. S6) which can be grouped into 121 structural clusters.

### Novel Circular Permutations of PDZ Domains.

**Fig. 4.**
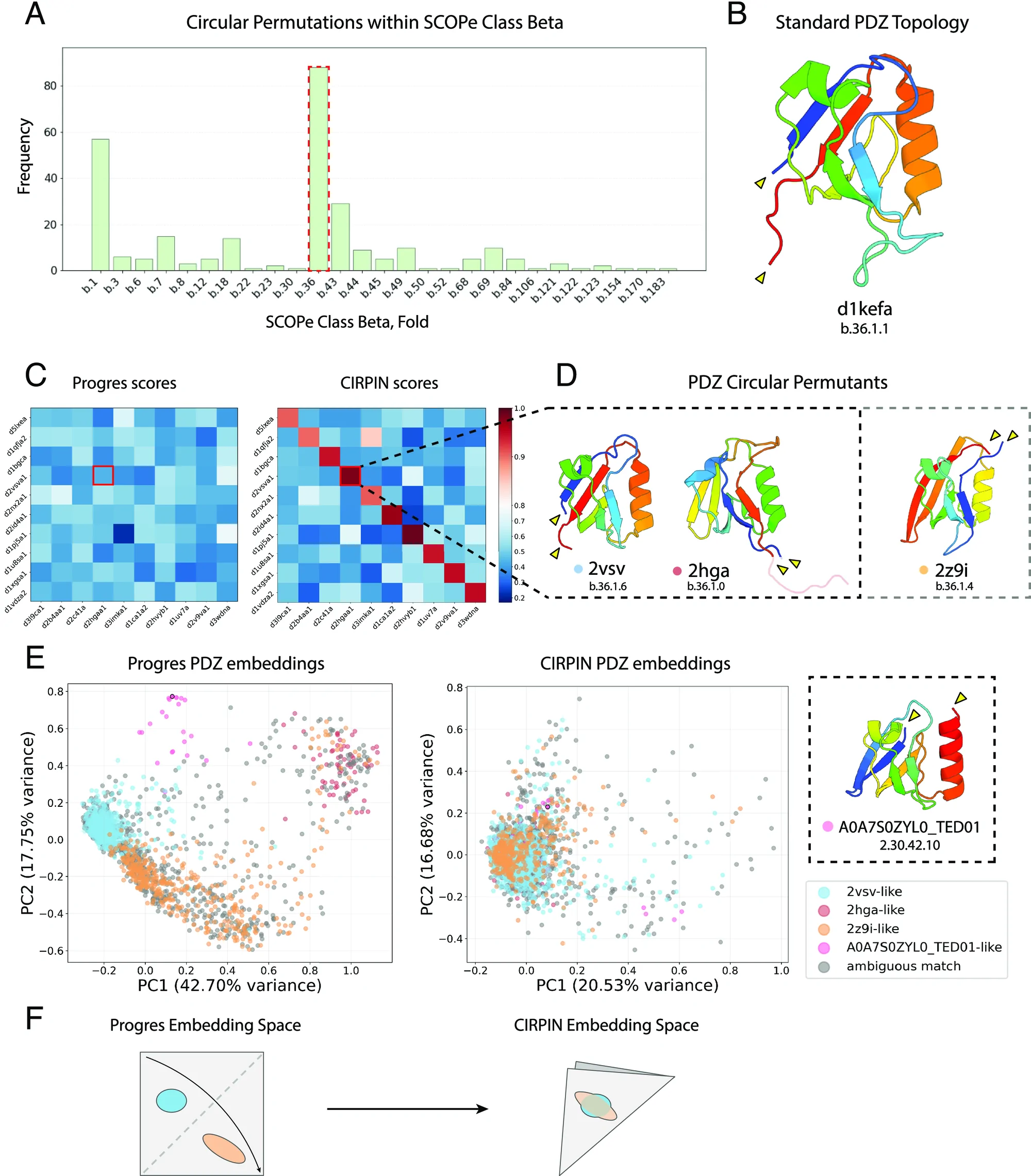
Discovery of novel circular permutations of PDZ domains by CIRPIN. (*A*) Number of circularly permuted pairs found in folds within the SCOPe class beta. Red highlights PDZ domain-like fold, b.36. (*B*) Prototypical PDZ fold. Termini are indicated by yellow triangles. (*C*) Heatmap of Progres/CIRPIN scores between representative protein pairs identified through Fig. 3. (*D*) Structures of three PDZ domains related by circular permutation, colored from N to C terminus. (*E*) PCA of AFDB-ClustR PDZ domains. Novel topology colored in pink, CATH label displayed below. (*F*) CIRPIN “folds” the Progres embedding space to bring together structures related by a CP.

To investigate the structural diversity of CPs identified by CIRPIN, we grouped them by the same SCOPe class and fold and plotted the number of occurrences (Fig. 4*A* and *SI Appendix*, Fig. S8). We then examined each class and identified folds that were overrepresented. An analysis of the beta class showed folds previously known to contain circular permutations [b.7 (35), b.12 (36), b.18 (13)] and revealed b.36 (PDZ domain-like) as a fold containing a high number of structures related by a CP (Fig. 4*A*). Based on these results, we further investigated the structures within the PDZ fold family.

PDZ is an acronym for three proteins first discovered to share the same domain: post synaptic density protein (PSD95), Drosophila disc large tumor suppressor (Dlg1), and zonula occludens-1 protein (zo-1). Since its initial characterization, the PDZ domain has been found to be widespread across metazoans, with over 400 proteins in *Homo sapiens* containing a PDZ domain (37). We examined PDZ domains from these three proteins and found they all shared the same N and C termini locations. We show one of these structures, the PDZ domain from PSD95/SAP90 (d1kefa) as the “standard” PDZ topology since most research has focused on PDZ domains exhibiting this topology (Fig. 4*B*).

Among the CPs identified by our search within the PDZ domain-like fold are two PDZ domains related by CP, PDB: 2vsv and PDB: 2hga. While previous research identified a CP of a PDZ domain in *Scenedesmus obliquus* (38) represented by the 2z9i structure, CIRPIN identifies an additional permutant, represented by the 2hga structure (Fig. 4*D*). We noted that 2hga contains a long C-terminal tail, which led us to wonder whether this feature contributed to its obscuration from Progres. However, truncating the C-terminal tail of 2hga did not improve Progres’s ability to detect homology between 2hga and 2z9i or 2vsv, while CIRPIN continued to assign a high score. For clarity in comparing the three topologies, we present the truncated version of 2hga. Comparison of the domain found in this study and ref. 38 reveals that there exist at least three distinct topological variants of PDZ domains, each related to one another by CP (Fig. 4*D*).

Given our discovery of circularly permuted PDZ domains within SCOPe we asked whether additional topologies may exist in larger databases. To address this, we took The Encyclopedia of Domains (TED) (39) annotated PDZ domains from the AFDB-ClustR (4,015 domains) and filtered for PDZ domains between 75 and 100 amino acids in length in order to remove poor quality annotations. This yielded 2,563 structures. We then plotted a PCA of the Progres and CIRPIN embeddings of these structures (Fig. 4*E*).

While analyzing the Progres PCA plot, we noticed a cluster of structures that appeared distinct from the three reference structures (Fig. 4*D*) we found in SCOPe. A representative structure from this cluster, shown in (Fig. 4*E*), constitutes another distinct PDZ circular permutant. We then colored the PCA plot according to which reference PDZ structure they best aligned to using TM-align. In some cases, a domain aligned well to multiple reference structures. If these scores were highly similar (within 0.025 of each other), we assigned these domains an “ambiguous match” label.

Owing to its circular permutation invariance, CIRPIN embeds CP-related proteins close to one another in space. Relative to Progres, this can be viewed abstractly as “folding” the Progres embedding space, whereby proteins related by a CP, once separated in space, now cluster together (Fig. 4*F*). Our approach, which compares the embeddings of a circularly invariant model (CIRPIN) to a noncircularly invariant model (Progres), allows for identification of CPs and investigation of the topologies present within a protein family.

### Identifying Novel Circular Permutations in AFDB-ClustR.

After verifying that our method can recover novel CPs from SCOPe, we applied the method to search for CPs in the AlphaFoldDB Cluster Representatives (AFDB-ClustR) (19). We first processed the AFDB-ClustR (∼2.7M representative structures) into individual domains (∼3.5 M domains) using Foldcomp (40) and The Encyclopedia of Domains (TED) annotations (39). Since this database is over two orders of magnitude larger than SCOPe, we used a more stringent filtering criteria; we filtered for pairs that have a Progres/CIRPIN score (ΔCP) of 0.5 and a CIRPIN score >0.8 (*Methods*). This resulted in 584,583,567 pairs, which we subsequently verified following the schematic shown in (Fig. 3*A*).

**Fig. 5.**
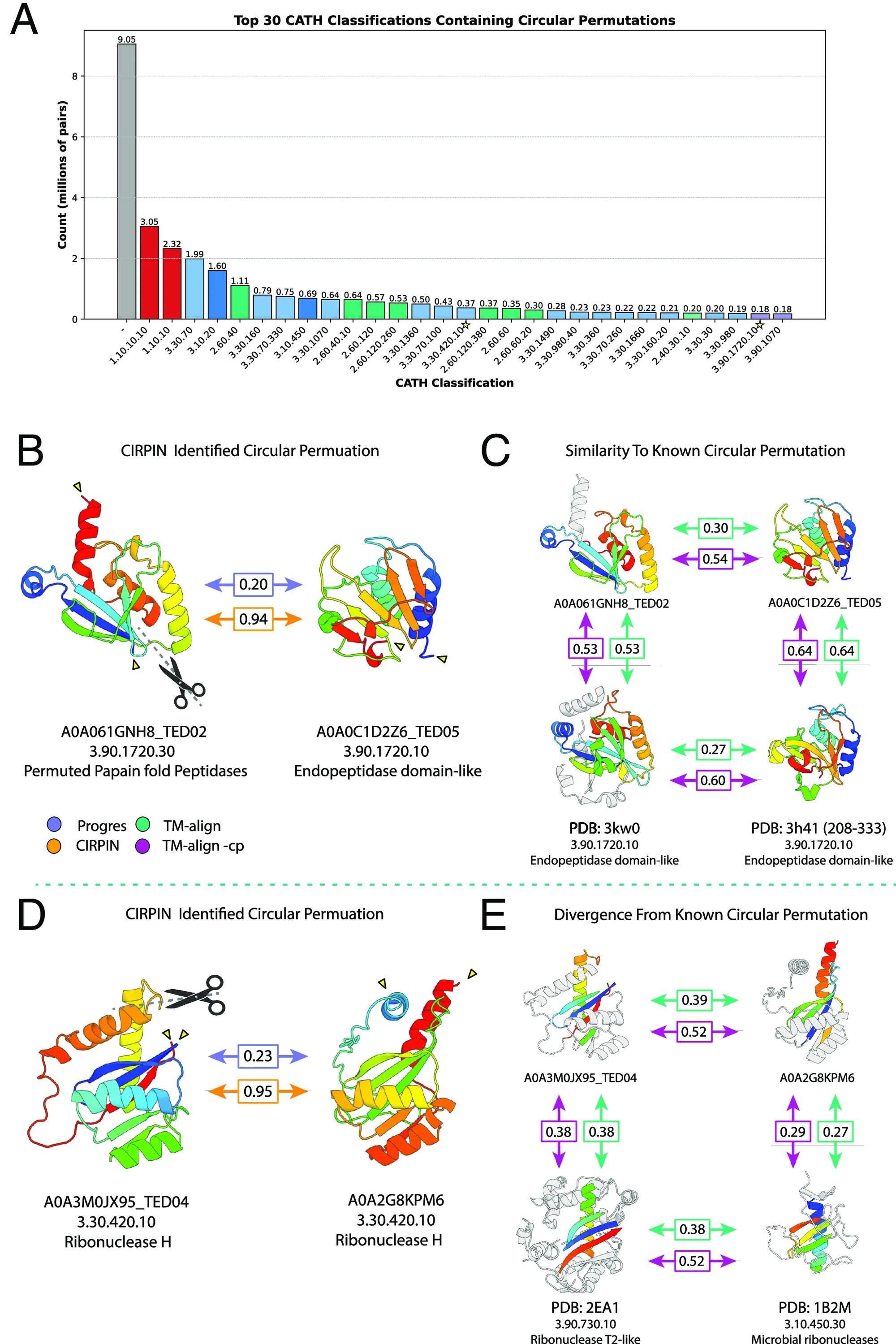
Circular permutations in the AFDB-ClustR identified by CIRPIN. (*A*) Distribution of CP structures according to TED CATH annotations (colored by CATH architecture). (*B*) Endopeptidase domains related by a CP. (*C*) Comparison of domains in (*B*) to a previously identified pair of endopeptidases. (*D*) Ribonuclease H-like domains related by a CP. (*E*) Comparison of domains in (*D*) with functionally related, though structurally divergent, ribonuclease T2-like (PDB 2EA1) and BECR fold (PDB: 1B2M) domains. The highlighted core motifs between the ribonuclease H domains and those from 2EA1 and 1B2M are below a TM-score of 0.5, even after accounting for a CP. Labels show the CATH level. Peripheral structural elements are colored gray to highlight the shared core structural motifs. Scores are calculated from the colored regions of the structures.

After filtering for pairs with a TM-align -cp score > 0.5 and a ΔTM score > 0 we obtained 18,301,678 pairs. From this dataset we identified 589,113 unique domains that have at least one structure related by a CP within the AFDB-ClustR. This dataset consists of structures from 845 unique CATH topologies. Among the most represented CATH domains are Winged helix-like DNA-binding domains (1.10.10.10) and Ubiquitin-like (UB roll) domains (3.10.20). However, 9 million pairs or about 50% of domains, lack CATH annotations (Fig. 5*A*). To further validate our dataset, we randomly sampled 100 pairs spanning more than 100 unique CATH annotations and manually assessed whether each pair appeared consistent with a CP relationship. We confidently classified 72 of the 100 pairs as circular permutants, 12 as unclear, and 16 as unrelated by a CP. The 12 marked as unclear were difficult to confidently call as cases of CP due to other structural changes. This corresponds to an estimated precision of 72% for our CP detection pipeline, including TM-align filters.

Clustering all 589,113 domains using the same parameters previously described resulted in 44,875 clusters compared to 121 clusters in CPDB after applying quality control (*SI Appendix*, Fig. S6).

Within our database, CIRPIN-DB (41), we found instances of CP that corroborate previous studies. We identified a pair of circularly permuted endopeptidases with structural similarity to those identified by ref. 42 (Fig. 5 *B* and *C*). In addition, we uncovered a CP between two ribonuclease H-like domains (Fig. 5*D*), which are key components of HIV-1 reverse transcriptase (43). We searched the literature for any instances of ribonucleases related by a CP and found one study which identified a CP between ribonuclease T2-like domains and the BECR fold RNAases (44) (Fig. 5*E*). Importantly, this earlier discovery relied on visual comparison of the two domains to reveal similarities in their core structural architecture since sequence and structure similarity tools were unable to identify homology (44). Comparing the core structures of the ribonuclease H–like domains reported here to those described by ref. 44 using TM-align yielded low TM-scores (0.38, 0.27) (Fig. 5*E*), highlighting CIRPIN’s sensitivity to detect putative remote homology that falls below commonly used structural similarity thresholds.

**Fig. 6.**
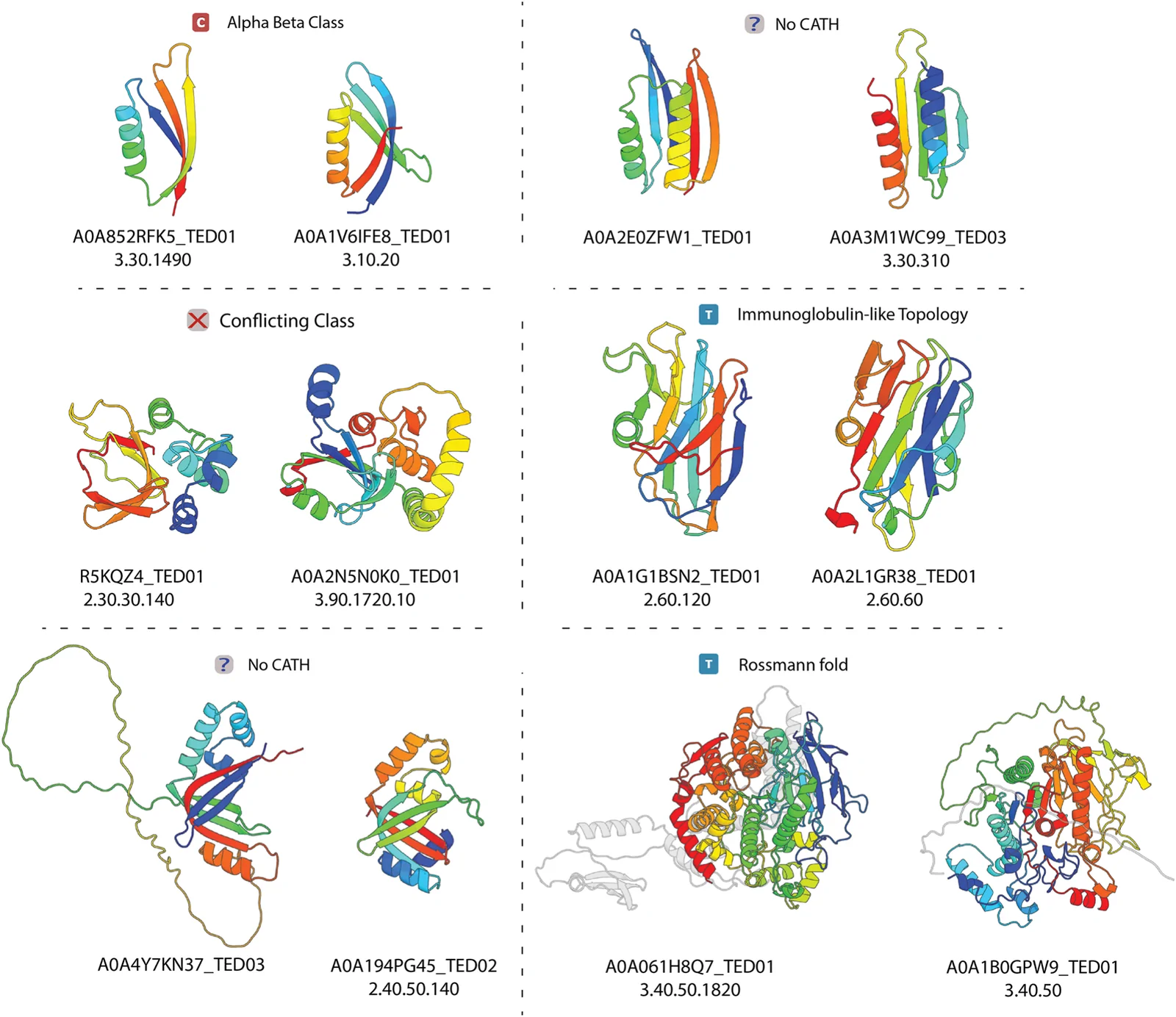
Novel circular permutations in the AFDB-ClustR. Icons above each pair indicate the CATH hierarchy shared between the two structures.

To broadly explore circular permutations in the CIRPIN-DB (41), we examined representative pairs of varying amino acid lengths and highlight several examples (Fig. 6). Many pairs are classified into different CATH classes, architectures, or topologies, or lack CATH annotations altogether (Fig. 6), demonstrating CIRPIN’s ability to identify CPs even when structural classifications are missing or inconsistent.

### CIRPIN Retrieves Protein Pairs Obscured by Rewiring and Insertions.

**Fig. 7.**
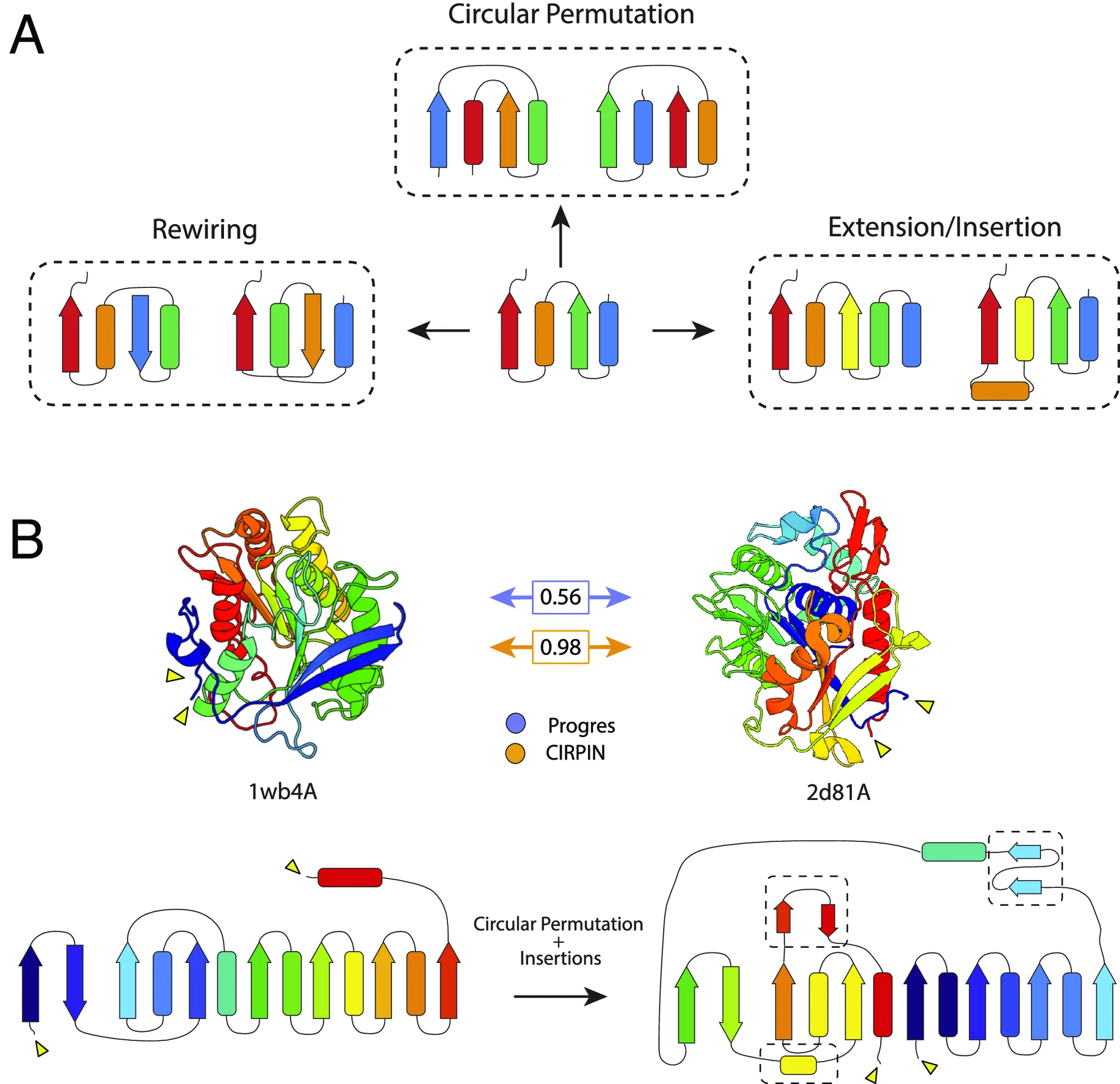
Examples of structural changes detected by CIRPIN. (*A*) Examples of structures related by a circular permutation, rewiring, extension, or insertion. (*B*) Example of a pair recovered from the “Circular Permutations” class which are related by a circular permutation and insertions. Yellow arrows highlight termini, dotted box highlights insertions.

**Fig. 8.**
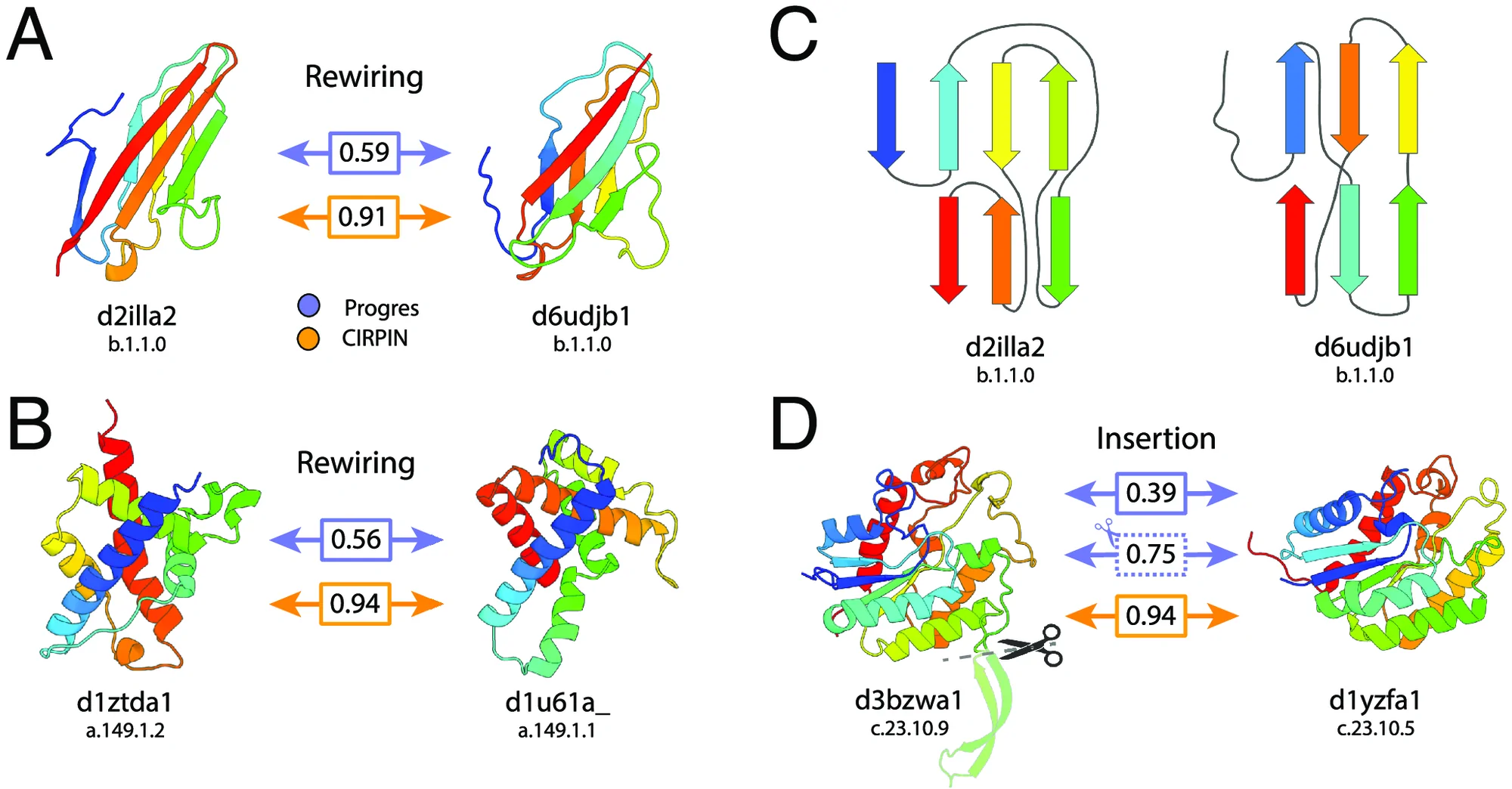
CIRPIN recovers proteins previously obscured by insertions and rewired secondary structure. (*A* and *B*) Proteins with similar architecture, different connectivity, recovered by CIRPIN. (*C*) Diagram showing differing connectivity of structures in (*A*). (*D*) Homologs previously obscured by an insertion. Boxes from *Top* to *Bottom*: (*Top*: Progres score on default structures), (*Middle* dotted: Progres score after removing insertion/extension), (*Bottom*: CIRPIN score on default structures).

Among the pairs that had the largest ΔCP scores, we noted many with high TM scores, but whose TM-score did not increase when applying the -cp flag (ΔTM=0). Within this class we observed structures which have the same architecture (arrangement of secondary structures in 3D space) but different secondary structure connectivity (Figs. 7*A* and 8*A*–*C*). We found that applying a CPO to one structure does not yield the same fold as the other; thus these structures are related by a rewiring of secondary structure elements that is distinct from CP. In Fig. 7*A*, the distinction between these relationships is shown. In cases of rewiring, internal connections may change, or be reconnected with one terminus, whereas in CP both termini must be connected and the new termini introduced elsewhere. Many proteins we identified as related by a CP in Fig. 3 contain additional structural changes, as highlighted by the example of polyhydroxybutyrate depolymerase (23), which in addition to CP has multiple insertions (Fig. 7*B*).

We investigated other cases with ΔCP scores and ΔTM=0 and found some are related by insertions or extensions. We hypothesized the differences in Progres and CIRPIN scores of these cases may be because Progres embeddings are highly sensitive to precise domain definitions. To test this, we removed the insertion from a representative example, rescored the domains and found this significantly increased the Progres score (Fig. 8*D*). Furthermore, we found that these alterations resulted in negligible changes in CIRPIN scores and therefore show a single CIRPIN score for this comparison.

The structures of one pair, shown in (Fig. 8*A*), are classified in the same SCOPe family, yet Progres still fails to find similarity between them. (Fig. 8*B*) shows a simplified diagram of these two structures to highlight the differences in connectivity. These results suggest that CIRPIN embeddings can be used to narrow the search space for pairs of proteins that may be related by more complex rearrangements beyond CP.

### Tertiary Motifs Emerge from CIRPIN Node Embeddings.

**Fig. 9.**
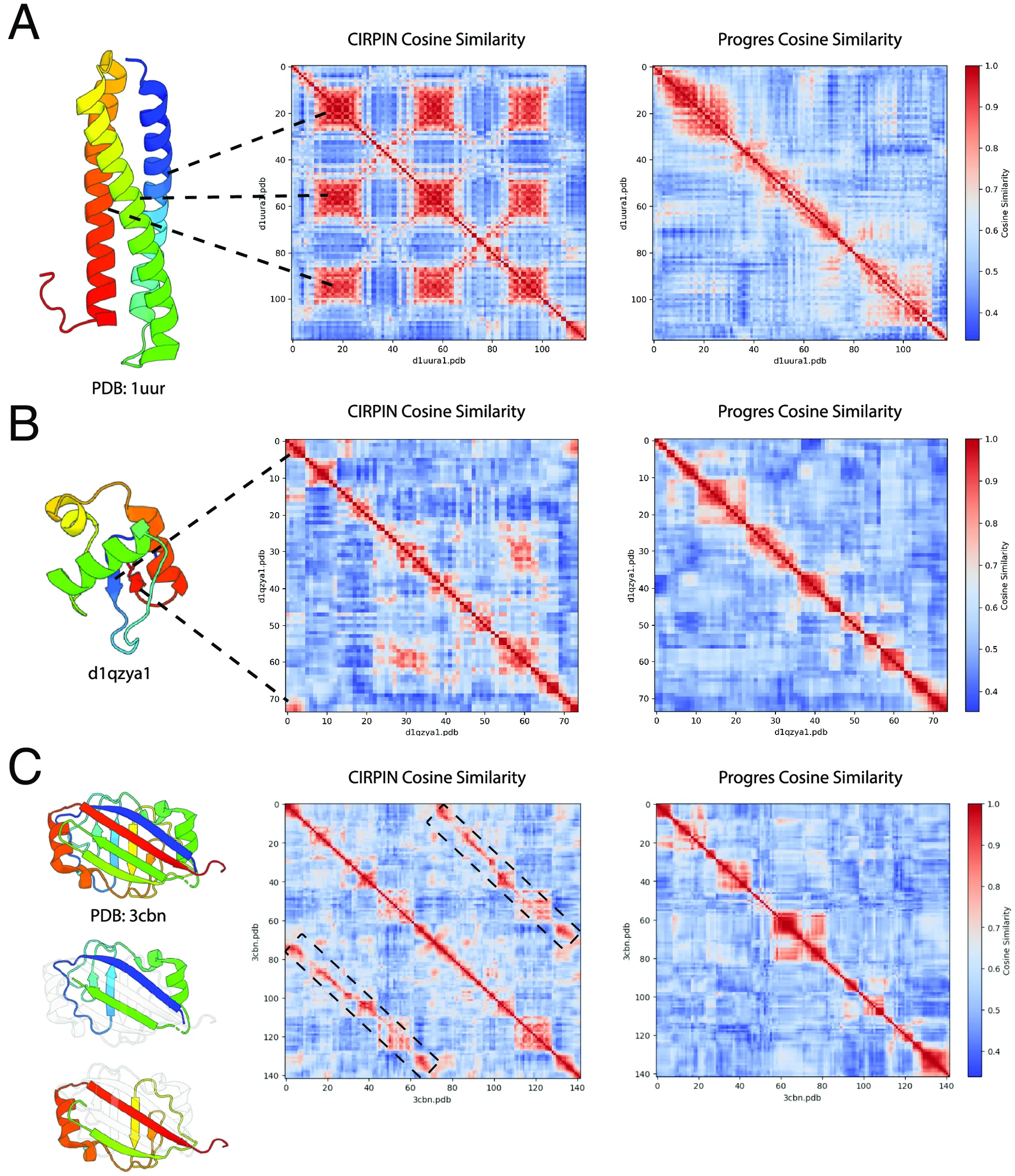
CIRPIN embeddings capture similarity between tertiary motifs that are absent in Progres embeddings. (*A*) CIRPIN and Progres node level embeddings for N terminus of PDB 1uur (self vs. self). Alpha helices show similarity to one another. (*B*) CIRPIN and Progres node level embeddings of QZ (self vs. self). N- and C-terminal beta sheets show high similarity. (*C*) CIRPIN and Progres node level embeddings of PDB 3cbn (self vs. self). Superimposable substructures of 3cbn show similarity in CIRPIN node-level embeddings (Black dashed regions).

To better understand how CIRPIN learns accurate embeddings for protein structure, we investigated the per node embeddings of proteins (Fig. 9*A*). Here we present the analysis of the N terminus of PDB 1uur, to more easily decipher differences between CIRPIN and Progres per node embeddings.

The N terminus of 1uur consists of three alpha helices of nearly equal length connected by two loop regions. Fig. 9*A* shows the result of extracting the per node embeddings of 1uur and plotting the cosine similarity between all nodes. The node-level heatmap for CIRPIN shows three regions of high cosine similarity that closely align to the alpha helices in the structure, indicating a correspondence between protein tertiary structure and latent space representations (Fig. 9). Analysis of QZ shows that CIRPIN finds similarity between residues within the two short N- and C-terminal beta sheets (Fig. 9*B*). These patterns of structural similarity are markedly absent in the same heatmaps generated by Progres (Fig. 9*B*).

To show whether CIRPIN’s ability to capture similarity within domains extends beyond simple recognition of alpha helices and beta sheets, we examined the more intricate structure of PDB: 3cbn. This protein has unique internal symmetry: The structure can be parsed into two nearly identical substructures that interlock like a handshake to form a single domain. These two substructures superimpose nearly perfectly (TM-score = 0.81) and are shown individually in (Fig. 9*C*). Analysis of the node-level embeddings shows that CIRPIN identifies these two substructures as highly similar, while the Progres heatmap lacks this signal (Fig. 9*C*). Taken together, these results indicate that by training on synCPs, CIRPIN learns features based on local context, while Progres may overrely on the positional information of residues when embedding protein structure. Given CIRPIN’s ability to capture substructure similarity, these embeddings could be used in future work to identify similar substructures or obtain explicit alignments.

## Discussion

Historically, discovery of proteins related by a CP has often come from researchers making careful observations of structural similarity on a protein of interest, as has been the case for the discovery of several proteins related by a CP (12, 35, 45). With CIRPIN, researchers can now systematically search across the protein structure databases for cases of CP. To lower the barrier for researchers to use our tool, we provide CIRPIN in a streamlined Google Colab notebook that allows for searching a protein of interest: https://github.com/aidenkoloj/CIRPIN/blob/main/CIRPIN.ipynb.

Using CIRPIN, we conducted a systematic search for circularly permuted proteins and generated the largest dataset to date of protein pairs related by a CP: CIRPIN-DB (41). Our dataset greatly expands the number of known CPs and will serve as a valuable resource for future studies investigating the evolution of circularly permuted proteins. In addition to CP, our identification of proteins related through extensions, insertions, or rewiring expands the set of structurally analogous pairs available for benchmarking challenging structure search tasks, addressing the severely limited size of current databases used for this task (46).

Among the novel pairs of CPs we identified are PDZ domains which we found to exist in four topological forms, each related by a CP. Although much has been published on the peptide specificity of the PDZ domain, only a few studies (47) have examined how a CP affects their folding and function. PDZ domains represent the most frequently inserted domain family (48) and characterization of their structural variants presented here will inform future work investigating the evolutionary radiation of this domain.

While CIRPIN identifies proteins related by a CP, it is important to note that such a relationship does not necessarily indicate that the two proteins descended from a common ancestral sequence. Studies investigating the mechanisms giving rise to CPs suggest that duplication of a gene followed by truncation of both the novel N and C termini [referred to as “creative destruction” (49)] is a highly probable route, though other mechanisms have yet to be entirely ruled out (50, 51). For instance, two structures could converge independently to the same fold, with differing N and C termini.

Filtering out very short protein pairs may help reduce cases of structural similarity arising from convergent evolution. We observed many pairs related by a CP that are short (∼60 to 80 amino acids) and consist of a small number (4 to 6) of secondary elements. Since there is a limited number of ways such a small number of elements can pack together into a stable fold, the likelihood these are a result of convergence is higher than for larger structures with complex arrangements of many secondary structures. On the other hand, some small domains related by circular permutation, such as the saposins and swaposins (45, 52) (composed of 4 alpha helices), have high sequence similarity, supporting a model of divergence from a duplicated ancestral sequence. Future work could examine specific examples identified by CIRPIN to unravel the evolutionary events leading to circular permutation and to potentially provide evidence for either convergent or divergent evolution.

CIRPIN adds to a growing suite of tools (5) that can identify structural similarity between proteins with nonsequential alignments. Using CIRPIN, we found many domains which lack CATH annotation, but are in fact structurally similar to known CATH families. In addition, we identified cases of CP where CATH annotations conflicted with one another. These examples demonstrate how CIRPIN can be used to unite proteins currently classified into different families, and potentially assign function to unannotated domains.

One important caveat of our current search method is the conservative ΔCP we used. Since CPs exist on a spectrum, our score difference likely limits our detection of more subtle CPs. Additional prefilters to reduce the search space, such as eliminating comparisons between domains of significantly different lengths, could enable less stringent scoring and capture relationships that were previously missed. Additionally, since CIRPIN was trained on the SCOPe database, it is unlikely to generalize well to protein domains that are highly divergent from those in the database. Furthermore, our analysis of the AFDB-ClustR may fail to detect instances of CP due to incorrect domain parsing. For example, due to large insertions, parsers may sometimes split domains into fragments.

Last, our method presents a compelling use case of semisynthetic data generation for addressing limitations of current structure search tools. While other methods for circular permutation detection have relied upon modifications to traditional alignment algorithms such as Smith-Waterman (15, 53), our method avoids explicit alignment in the first step which makes large-scale search for CPs feasible.

To encourage further investigation, we provide a complete list of CPs identified by CIRPIN [CIRPIN-DB (41)] and the model weights and training script at: https://github.com/aidenkoloj/CIRPIN (54). CIRPIN is accessible through an online search interface at https://cirpin.solab.org/.

## Methods

To detect proteins related by a CP, we introduce a learning framework built on an existing graph neural network architecture with our proposed synthetic circular permutation operation (synCP). Our method, CIRPIN, learns embeddings that are robust to permutations in sequence connectivity while preserving essential 3D structural information of proteins. In this section, we describe how proteins are represented, formalize our synCP operation, and describe the training, evaluation, and search pipelines. In addition, we provide justifications for our choice of score thresholds and cutoffs.

### Protein Structure Representation.

We represent each protein structure as a graph G=(V,E) where the vertices correspond to ${\text{C}}_{\alpha }$ atoms extracted from the protein backbone and the edges are constructed based on spatial proximity between ${\text{C}}_{\alpha }$ atoms. Specifically, we connect two nodes $v_{i}$ and $v_{j}$ with an undirected edge $(v_{i},v_{j})\in E$ if their corresponding ${\text{C}}_{\alpha }$ atoms are within 10 Å of each other.

For a protein containing *n* residues, we define the ordered sequence of ${\text{C}}_{\alpha }$ coordinates as $P={\left\{p_{i}\right\}}_{i=1}^{n}$, where $p_{i}\in R^{3}$ denotes the spatial coordinates of the *i*-th ${\text{C}}_{\alpha }$ atom. The indexing follows the natural N-to-C terminal direction ality of the protein chain, such that $p_{1}$ corresponds to the N terminus and $p_{n}$ to the C terminus.

As described in ref. 17, each vertex $v_{i}\in V$ is associated with a feature vector $f_{i}=\left[\rho_{i},\tau_{i},s_{i}\right]$ that encodes both local structural properties and sequential information where:

- $\rho_{i}=\frac{|\alpha_{i}|}{\max\left\{|\alpha_{1}\left|,\cdots ,\right|\alpha_{n}|\right\}}$ for $\alpha_{i}=\left\{p_{k}:{\left\|p_{k}-p_{i}\right\|}_{2}\leq 10\right\}$ is the normalized local density within a 10 Å neighborhood.
- $\tau_{i}=\left[\begin{array}{c} \tau_{N}\\ \tau_{C} \end{array}\right]\in \left\{\left[\begin{array}{c} 1\\ 0 \end{array}\right],\left[\begin{array}{c} 0\\ 1 \end{array}\right],\left[\begin{array}{c} 0\\ 0 \end{array}\right]\right\}$ where the vectors correspond to whether it is N-terminal, C-terminal, or neither.
- $s_{i}\in R^{64}$ is a sinusoidal positional encoding (55).

### Synthetic Circular Permutation.

To test a model’s performance on identi fying similarity between proteins related by a CP, we introduce the concept of synthetic circular permutations (synCPs). For any protein of length *n*, there are *n* possible circular permutations. We generate synthetic circular permutations of a given protein by circularly reordering the protein’s ${\text{C}}_{\alpha }$ coordinates.

Given the original ordered sequence of ${\text{C}}_{\alpha }$ coordinates P, a *k*-synthetic circular permutation is defined as

[1] $$\begin{array}{c} P_{k}=\left\{p_{k+1},\ldots ,p_{n},p_{1},\ldots ,p_{k}\right\}. \end{array}$$

This operation can be implemented in PyTorch using $P_{k}=\mathrm{torch.roll}\left(P,n-k\right)$.

Two structures related by synCP will have nearly identical graphs, with edges differing only at the original and new N and C termini due to the breaking and formation of terminal connections. However, the positional embeddings $S_{i}$ at each node will be circularly permuted according to the new sequence ordering. The terminal indicator feature $\tau_{i}$ will also change to reflect the new termini positions. Due to the fact that these graphs do not necessarily correspond to real circular permutations that occur in nature, we refer to them as “synCPs.”

### CIRPIN Architecture and Training.

CIRPIN builds upon the Progres model architecture, which uses an E(n)-equivariant graph neural network (18) and supervised contrastive loss to embed protein structures (56) (Fig. 1*B*).

We add a synCP generation module that creates random circular permutations during training (Fig. 1*A*). We extend the construction of positive and negative pairings of proteins described in ref. 17, where positive pairings are computed by taking proteins in the same SCOPe family. In each training iteration, we sample a structure and perform a random synCP on that structure. This operation is represented by the ouroboros, an ancient symbol of rebirth, on Fig. 1*A*. Over the course of many epochs, the model sees a diverse range of synCPs of each protein in the training set. Using supervised contrastive loss, proteins within the same SCOPe family are brought closer together in latent space and more distantly related proteins are moved far apart. However, upon implementing CIRPIN, distantly related proteins sharing higher SCOPe classification, such as fold or superfamily, may now be pushed closer in latent space.

We trained the model for 500 epochs and used the Progres validation set to monitor performance on recovering domains in SCOPe from the same fold, superfamily, and family, as described in ref. 3. We selected the model with best validation set performance for all further use. After training the model, database search is performed rapidly by ranking the cosine similarity between the embedding of an input protein against all proteins in a pre-embedded database.

### Foldseek Clustering.

We performed structural clustering using Foldseek easy-cluster with the parameters used previously in ref. 19: alignment type 1 (TM-align), set cover clustering mode, an E-value threshold of 0.01, a coverage of 0.9 (coverage mode 0), and a TM-score threshold of 0.5.

### Structural Alignment with TM-Align.

We use TM-align, a structural align ment algorithm based on the TM score metric (25) and commonly used as a benchmark metric in competitions such as the Critical Assessment of protein Structure Prediction (CASP) (57) as a sensitive, but computationally expensive, structural alignment method. A TM score of <0.17 is considered to represent random or no significant structural similarity, whereas a score >0.5 suggests the proteins are highly similar as it would take at least 1.8 million random structural matches so that one structure match reaches a TM-score ≥ 0.5 (25). We therefore use 0.5 as a cutoff for CP retrieval. TM-align returns two scores, normalized by either the query or target length. In all analyses we take the minimum score returned by TM-align, since we perform our search on single domains and thus seek a score reflecting global similarity between two structures.

TM-align depends upon dynamic programming to generate optimal alignments. Consequently, the algorithm is sensitive to sequence order, and thus to aligning circular permutants. However, an optional -cp duplicates the query structure to allow the alignment to wrap around, enabling more accurate alignment of circular permutants. Variations of this general approach, “duplicate and align,” have been used in several previous works (14–16).

Since TM-align CP also reports high scores for protein pairs unrelated by CP, it is necessary to compute both the TM-align and TM-align CP score. By taking the difference, ΔTM, we can identify pairs in which allowing for circular permuta tion resulted in an improved alignment. We calculated the ΔTM score for the test set of CPs and found that pairs of CPs had the highest ΔTM scores (*SI Appendix*, Fig. S9). However, we did note that pairs unrelated by CP can have slightly improved alignments as well (ΔTM>0) (*SI Appendix*, Fig. S9).

### AFDB-ClustR Threshold Determination.

Since Progres and CIRPIN were trained on SCOPe, metrics used on the training set may not be appropriate for unseen data. As such, we reassessed our choice of thresholds to identify putative CPs. First, we filter for pairs with a CIRPIN > 0.8, since we expect any pair related by circular permutation to have high similarity by CIRPIN.

Next, we set a score difference between CIRPIN and Progres (ΔCP) and performed the following analysis to determine a reasonable ΔCP. We varied $\Delta CP\in \left\{0.2,0.3,\ldots ,0.7\right\}$, sampled 10,000 pairs at each cutoff and measured the fraction passing TM-align structural validation (TM-align -CP > 0.5 and ΔTM>0) (Fig. 3 and *SI Appendix*, Table S1). At low ΔCP cutoffs the percentage of pairs with high ΔTM diminishes, and below ΔCP=0.4, no pairs have ΔTM>0.3 (*SI Appendix*, Table S1). This indicates that the pairs selected at low ΔCP are likely related through either very subtle circular permutation, wherein the termini move very little, or possibly other small structural deviations. Higher ΔCP cutoffs retrieve a higher percentage of pairs with ΔTM>0.2, which is more likely to represent clearer instances of circular permutation. In addition, cutoffs below 0.5 are computationally infeasible: A cutoff of 0.4 alone yields 8.9 billion candidate pairs (*SI Appendix*, Table S1), making exhaustive TM-align validation prohibitively expensive in both runtime and compute resources, and therefore impractical for large-scale structural screening. We therefore choose ΔCP=0.5 as our initial filter.

## Supplementary Material

Appendix 01 (PDF)

## Data Availability

Database of protein pairs related by circular permutation, training data and model weights, and analysis scripts for figures have been deposited in https://github.com/aidenkoloj/CIRPIN (54) and https://huggingface.co/datasets/aidenkzj/CIRPIN/tree/main (41). All other data are included in the manuscript and/or *SI Appendix*.
