# Supplementary material for: Systematic discovery of circular permutations across the protein universe using CIRPIN
Source: Proc Natl Acad Sci U S A. 2026 Sep 4;123(36):e2605178123. doi: 10.1073/pnas.2605178123 (PMC13552848; doi:10.1073/pnas.2605178123)
Supplement: Supplementary file 1 [file pnas.2605178123.sapp.pdf]

Supporting Information for

Systematic discovery of circular permutations across the protein universe using CIRPIN

Sergey Ovchinnikov
E-mail: so3@mit.edu

This PDF file includes:

Supporting text
Figs. S1 to S11
Table S1
SI References

Supporting Information Text

(a) All vs all scores for d2dk8a1 vs d1qzya1: CIRPIN (orange), Progres (blue), TM-vec (red) models

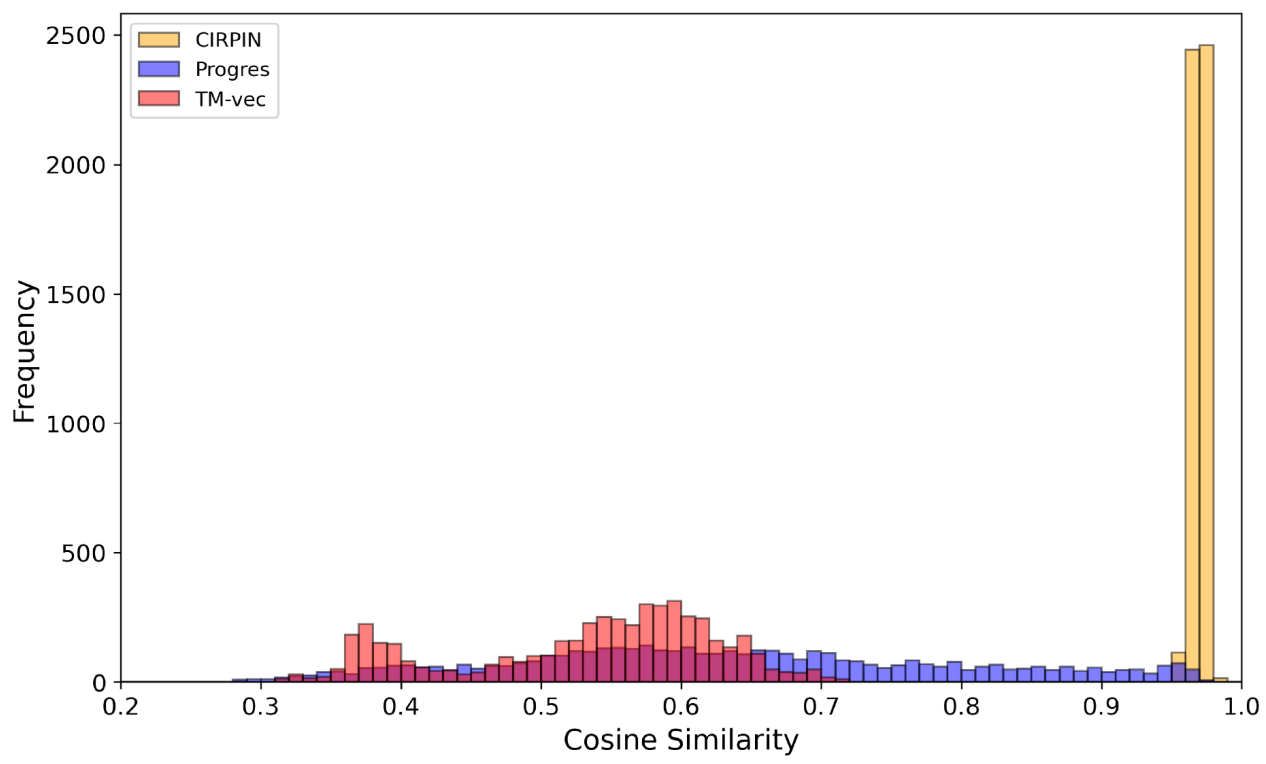

(b) Distributions of TM-align scores for synCPs of d1qzya1 and d2dk8a1

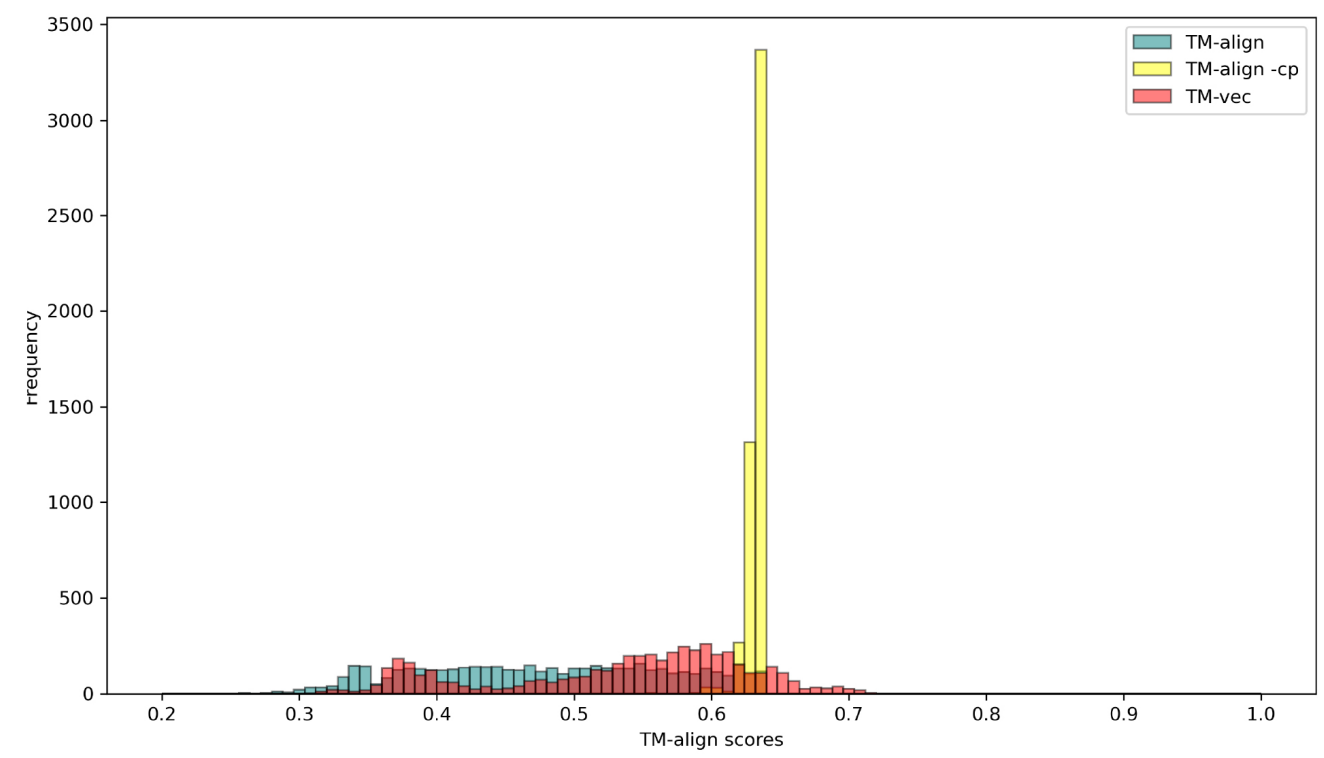

(c) Minimum synCP score

CP Pair	CIRPIN	Progres	TM-Vec
d1qasa2 & d1rsya1	0.975407	0.412015	0.658488
d1puja_ & d1zbda_	0.883974	0.342954	0.424968
d1cida1 & d1dcea2	0.809480	0.388181	0.392981
d1vhra_ & d1phra_	0.782694	0.364720	0.403653
d1mkza1 & d1jbwa1	0.742505	0.343785	0.398089
d2dk8a1 & d1qzya1	0.953684	0.267652	0.310037
d1iu9a_ & d1h0ra_	0.736251	0.379321	0.479205
d1qdma1 & d2gtga_	0.974847	0.674746	0.726851
d1booa_ & d1g38a1	0.847141	0.477426	0.469344
d3cnaa_ & d2pela_	0.989974	0.461548	0.632790
d1p8ja1 & d1ca1a2	0.974471	0.413952	0.442830

(d) Range of synCP score distributions

CP Pair	CIRPIN	Progres	TM-Vec
d1qasa2 & d1rsya1	0.019764	0.583527	0.227358
d1puja_ & d1zbda_	0.075348	0.630037	0.234344
d1cida1 & d1dcea2	0.070560	0.571518	0.281679
d1vhra_ & d1phra_	0.055206	0.572321	0.118635
d1mkza1 & d1jbwa1	0.093402	0.618927	0.161224
d2dk8a1 & d1qzya1	0.027627	0.705396	0.409501
d1iu9a_ & d1h0ra_	0.064763	0.581464	0.150846
d1qdma1 & d2gtga_	0.020889	0.322977	0.198557
d1booa_ & d1g38a1	0.067540	0.483792	0.246818
d3cnaa_ & d2pela_	0.009133	0.537570	0.334193
d1p8ja1 & d1ca1a2	0.013656	0.577414	0.225306

Fig. S1. (a-d) Comparison of Progres (1), CIRPIN, and TM-vec (2) performance on scoring a benchmark dataset of circular permutant pairs. (a) Representative distribution of synCP scores for circular permutants QZ and K8. (b) Minimum synCP scores taken from distributions shown in (a) for all pairs of circular permutants; higher scores (indicating structural similarity) are highlighted in yellow. (c) Range of synCP score distributions for Progres, CIRPIN, and TM-vec; smaller ranges (indicating greater invariance to circular permutation) are highlighted in yellow. Plotting the distributions across all synCP combinations reveals each model's sensitivity to positional reordering. We use the distribution range as a measure of invariance to circular permutation, with smaller ranges reflecting greater invariance, and also report the minimum score to capture cases where a model spuriously indicates dissimilarity due to reordering.

Table S1. Estimated numbers of putative circularly permuted protein pairs passing different △TM thresholds alongside total candidate pools as a function of the △CP cutoff.

△CP	Total Candidates	Estimated CPs at △TM cutoffs			
		△TM > 0.0	△TM > 0.1	△TM > 0.2	△TM > 0.3
0.2	381.9B	611.1M	191.0M	0	0
0.3	71.8B	229.8M	86.2M	14.4M	0
0.4	8.9B	63.5M	33.1M	4.5M	0
0.5	756.9M	19.2M	10.9M	2.9M	75.7K
0.6	41.9M	2.5M	1.4M	343.9K	12.6K
0.7	833.4K	84.8K	54.4K	11.8K	500.0

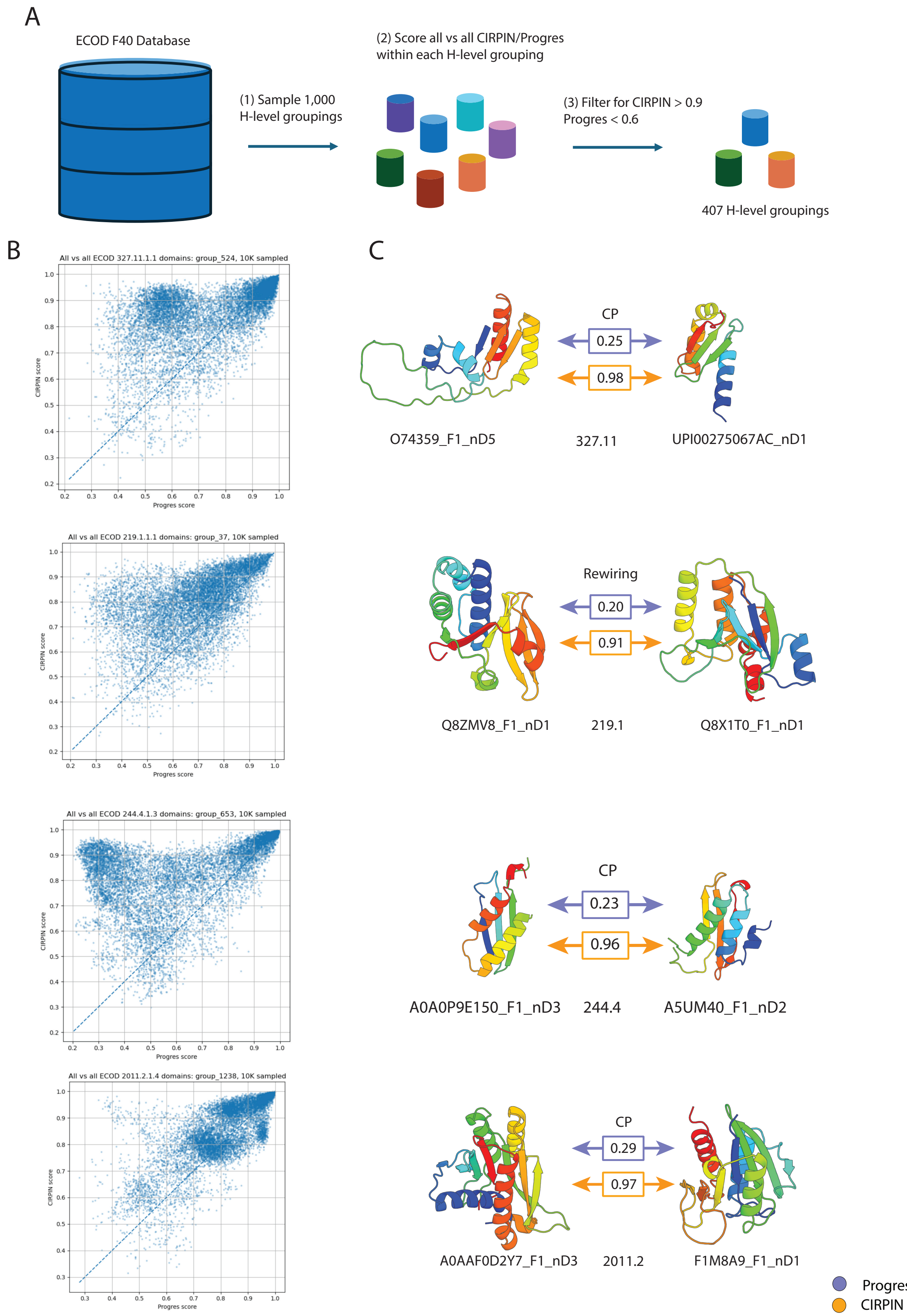

Fig. S2. Recovering homologous proteins in ECOD. (A) Identification of ECOD H-groups with homologous proteins detected by CIRPIN, missed by Progres. (B) Representative pairs from (A). Pairs were chosen from the 407 groups after additional filtering for largest ΔCP. ECOD names and ECOD X,H-level classifications displayed below structures.

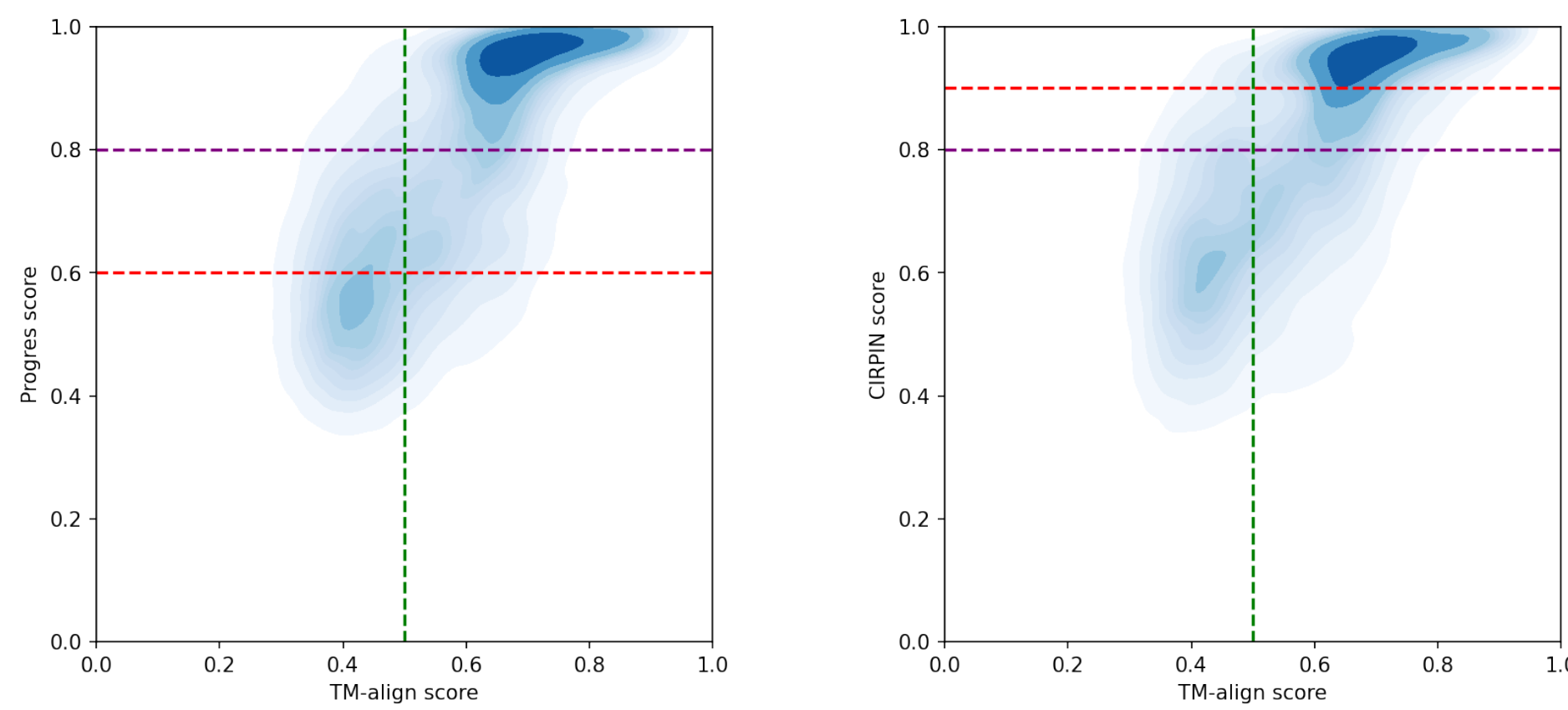

Fig. S3. CIRPIN/Progres cutoffs determination for SCOPe. 400 test domains were searched against the SCOPe database and the top 200 hits by TM-align were retained and plotted. Areas of dissimilarity fall below 0.6 for Progres and areas of similarity are above 0.9 for CIRPIN.

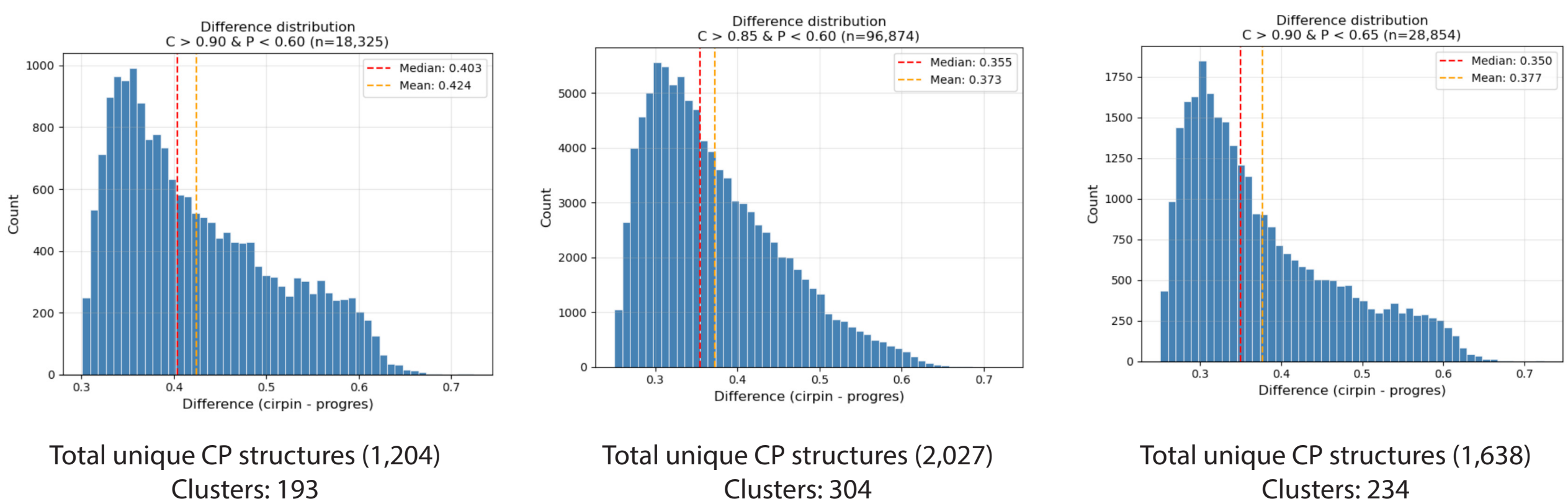

Fig. S4. Clustering results for three different CIRPIN/Progres thresholds

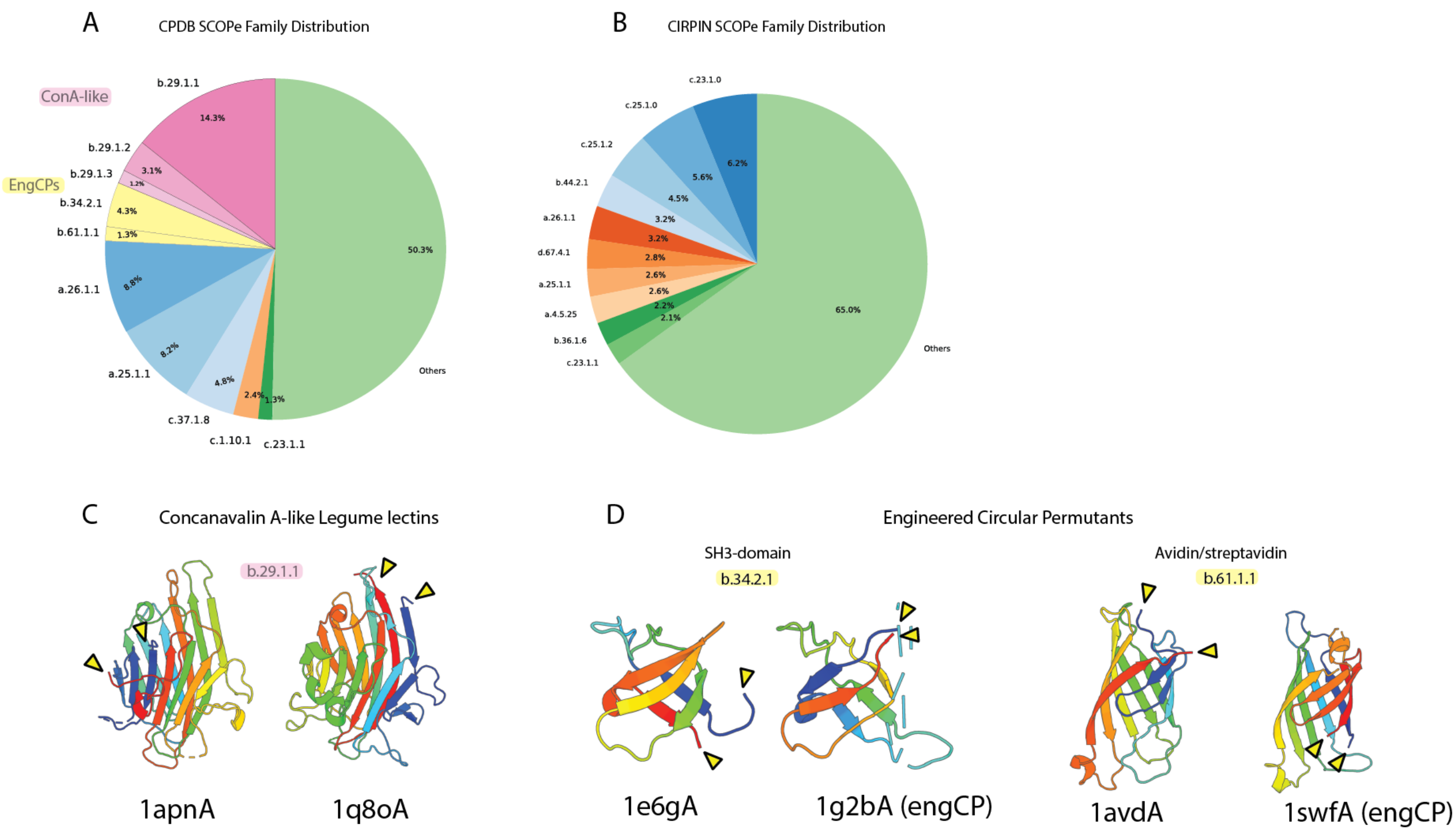

Fig. S5. SCOPe families represented in the CPDB and CIRPIN datasets. (A) CPDB SCOPe families. ConA-like families highlighted in pink. Families containing engineered CPs (engCPs) highlighted in yellow. (B) CIRPIN SCOPe families. (C) ConA-like structures. (D) Engineered circular permutations present in the CPDB.

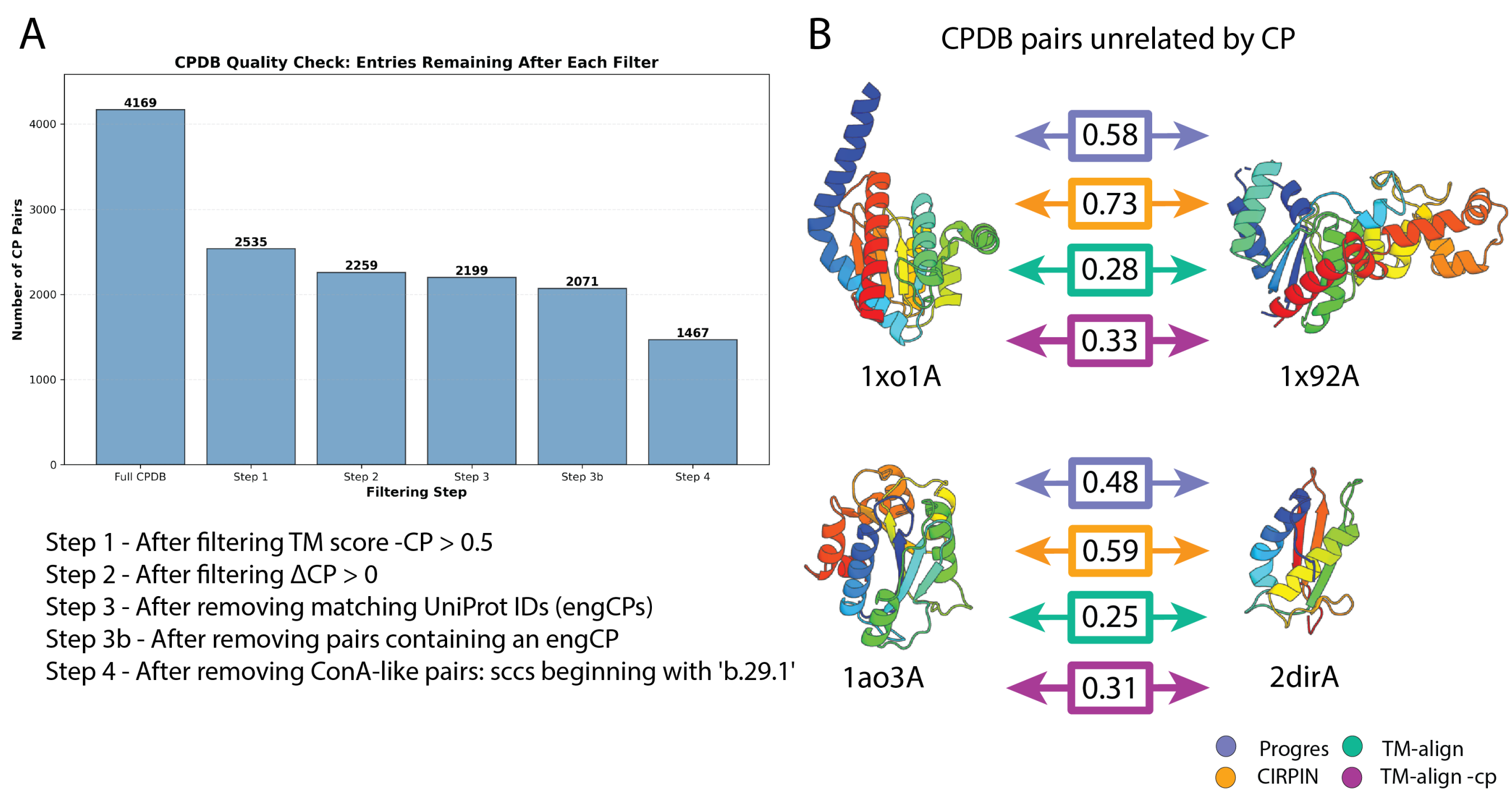

Fig. S6. Quality control of CPDB. (A) Filters (steps 1,2) used for CIRPIN applied to CPDB. Removal of overrepresented ConA-like structures and engineered (engCPs). (B) Examples of pairs present in the full CPDB that do not appear related by circular permutation.

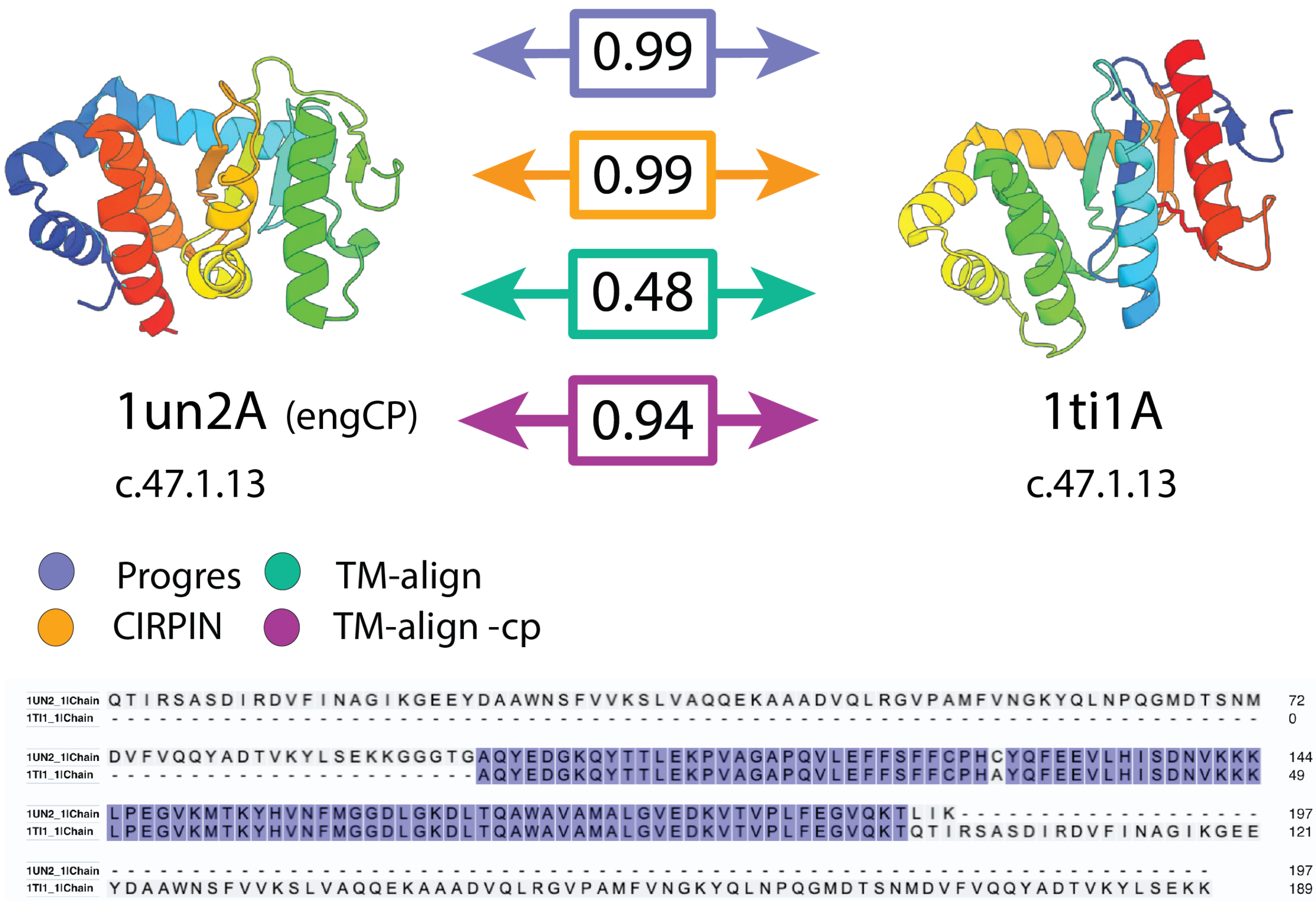

Fig. S7. Example of engineered circular permutant of DsbA (PDB: 1UN2) present in the CPDB. Sequence alignment shown below (half aligned due to CP). Due to classification in the same SCOPe family, the CIRPIN/Progres scores are identical and this pair is excluded from CIRPIN hits.

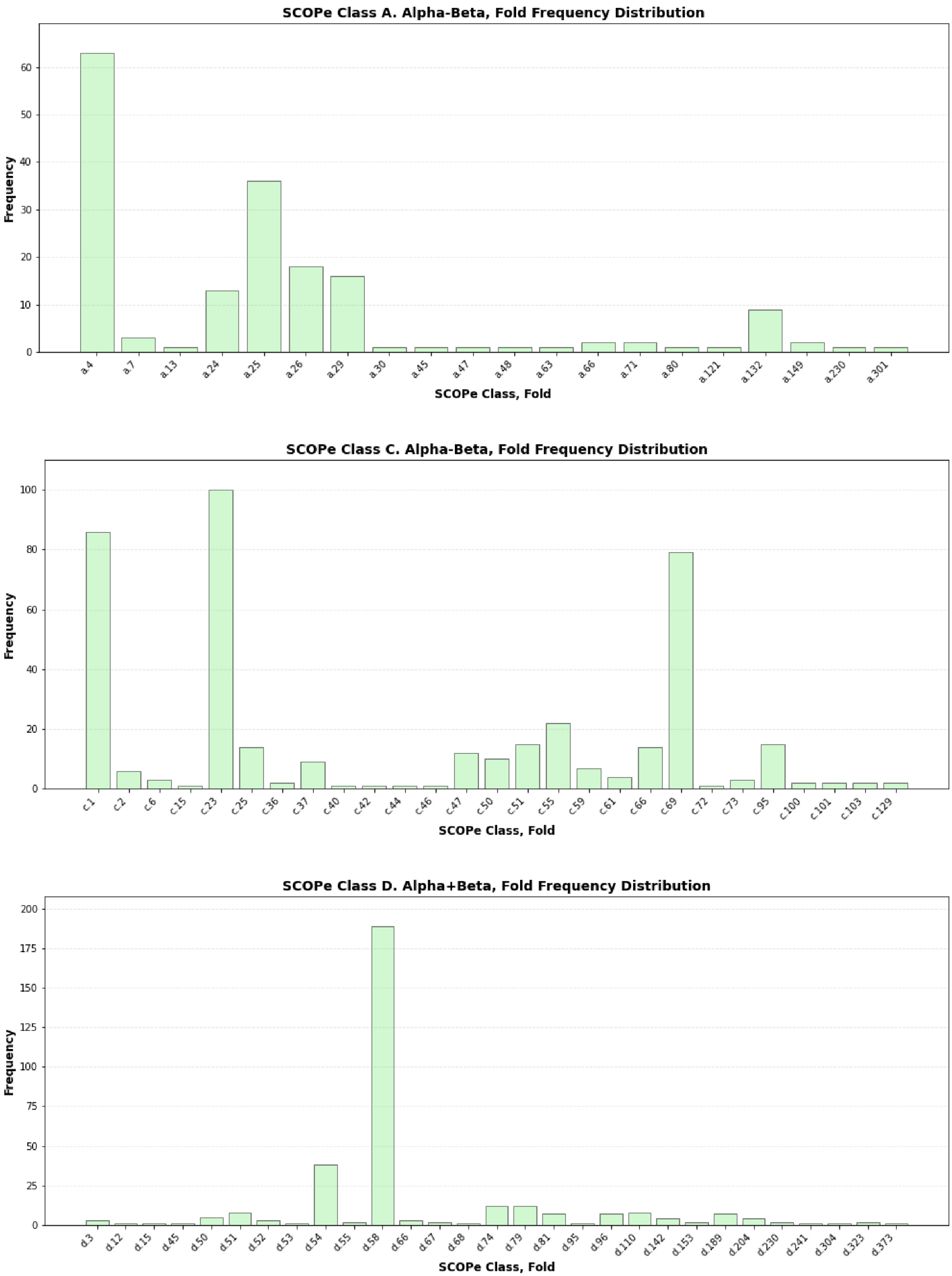

Fig. S8. Distribution of SCOPe CPs by SCOPe class and fold

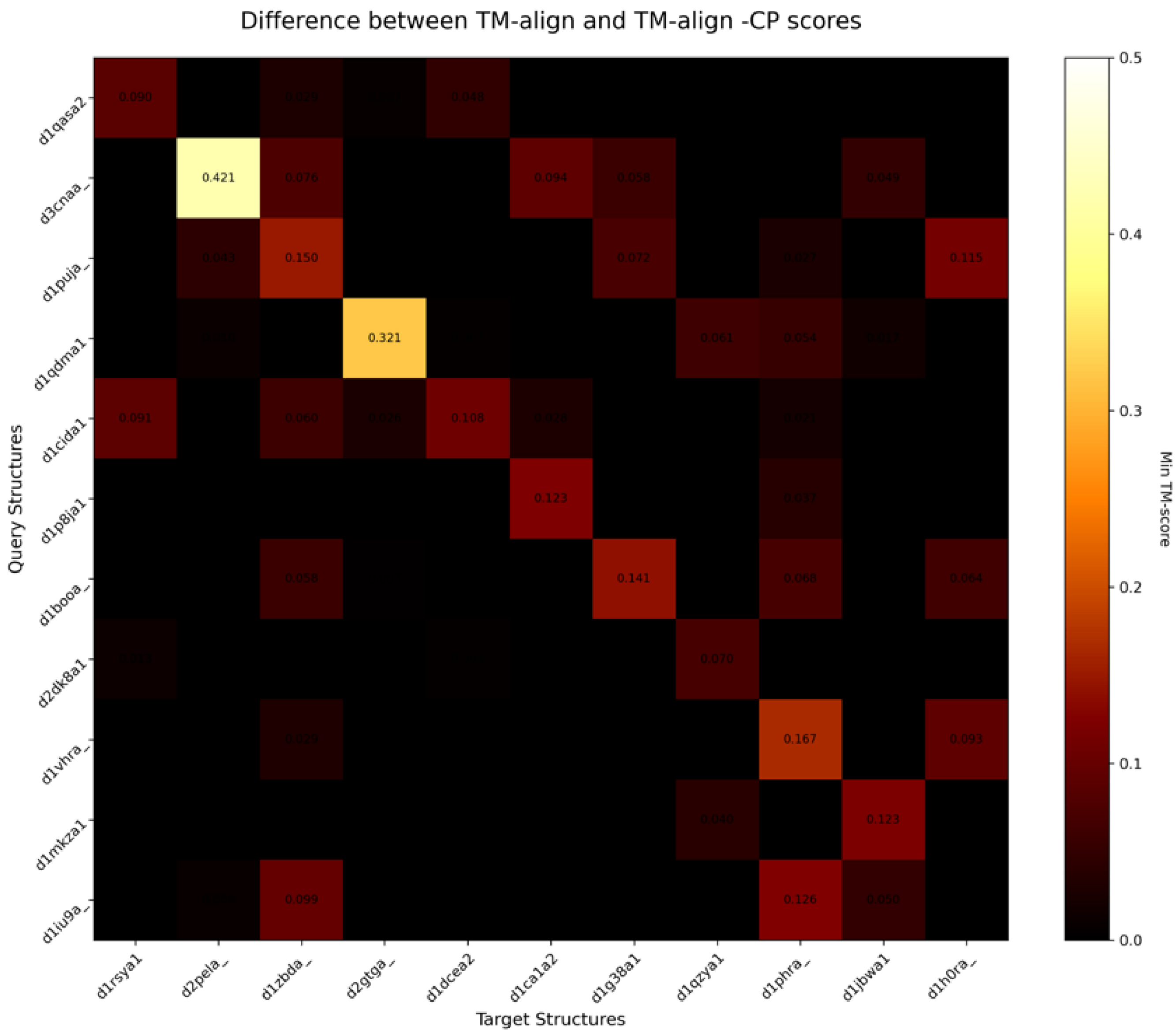

Fig. S9. ΔTM scores (difference between TM-align and TM-align -cp scores) for the test set of CPs.

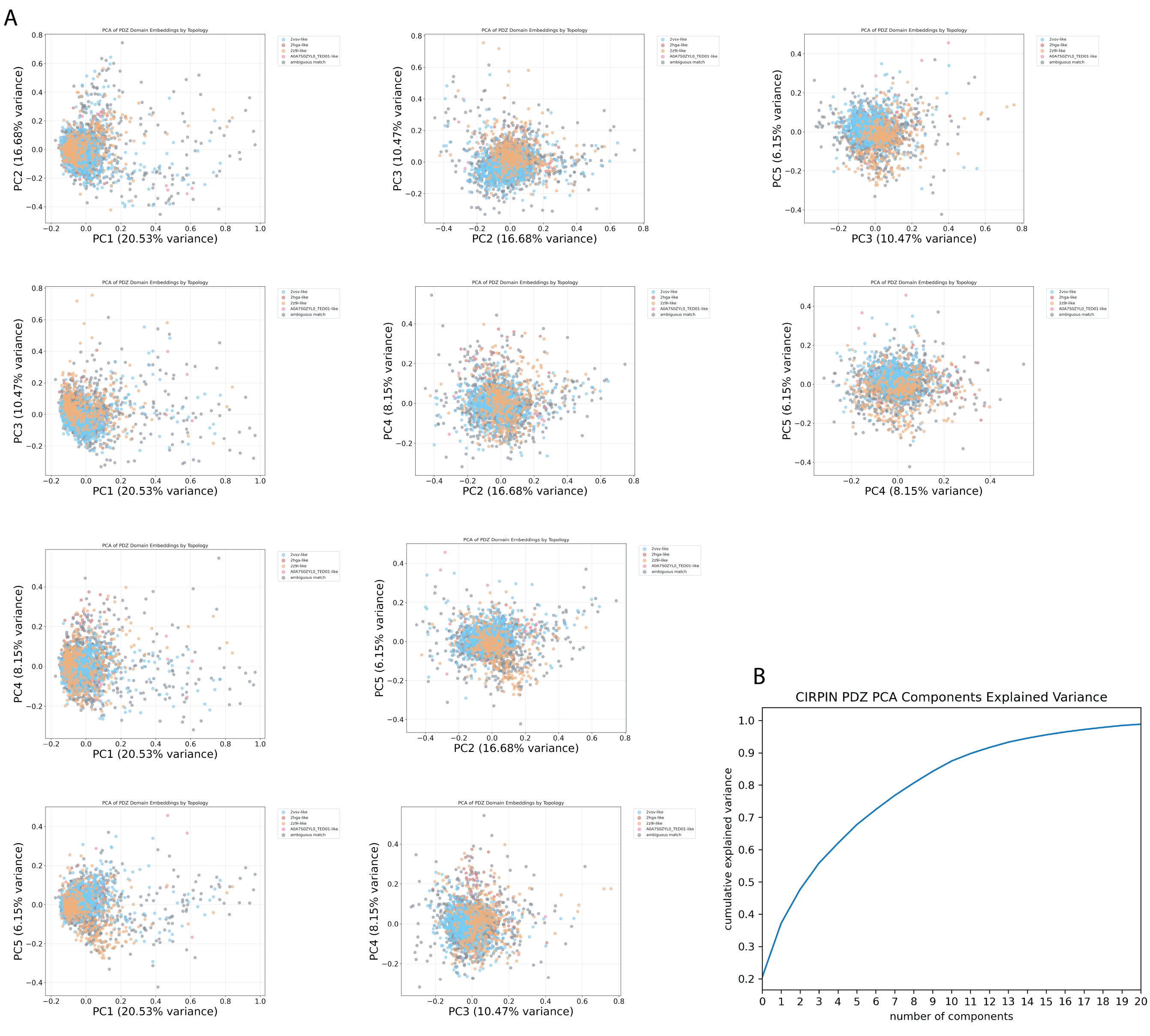

Fig. S10. PCA of PDZ domains embedded by CIRPIN (A) Principal component comparisons (B) Cumulative variance explained by principal components

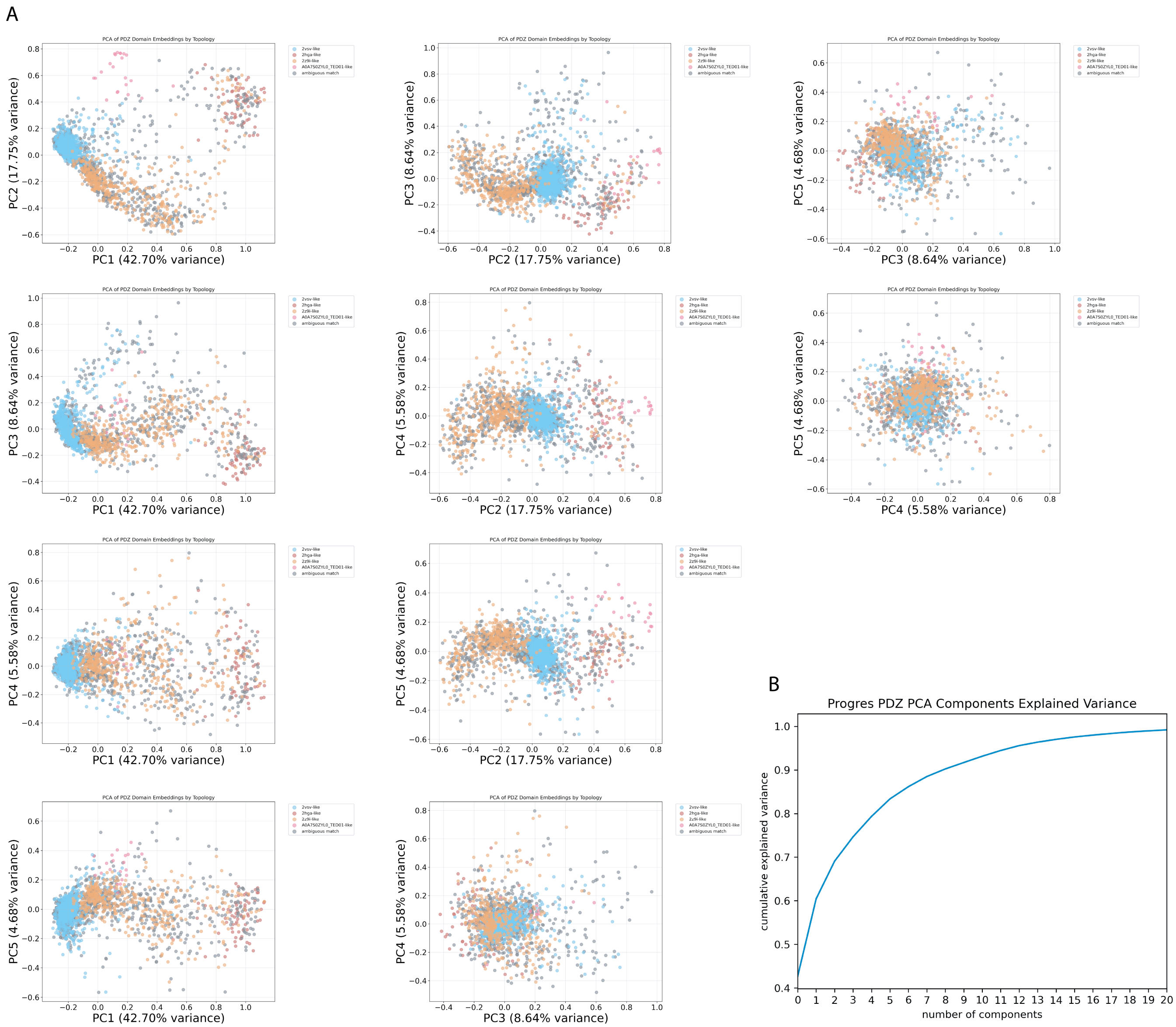

Fig. S11. PCA of PDZ domains embedded by Progres (A) Principal component comparisons (B) Cumulative variance explained by principal components

References

1. JG Greener, K Jamali, Fast protein structure searching using structure graph embeddings. *Bioinforma. Adv.* **5**, vbaf042 (2025).
2. T Hamamsy, et al., Protein remote homology detection and structural alignment using deep learning. *Nat. biotechnology* **42**, 975–985 (2024).
